# Silence on the plate: revisiting the enigma of *Mycobacterium leprae* cultivation

**DOI:** 10.3389/fmicb.2025.1708557

**Published:** 2025-12-03

**Authors:** Alfonso Gotor-Rivera, Natalia Gutiérrez-Casado, Lucrecia Acosta-Soto

**Affiliations:** 1 Department of Dermatology, University Hospital “12 de Octubre”, Madrid, Spain; 2 Institutional Library, University Hospital “12 de Octubre”, Madrid, Spain; 3 Area of Parasitology, University “Miguel Hernández”, Elche, Spain; 4 Fontilles Sanatorium, Alicante, Spain

**Keywords:** *Mycobacterium leprae*, culture media, *in vitro*, axenic, cell cullture, culture

## Abstract

**Introduction:**

*Mycobacteriumleprae* remains uncultivable in axenic media, a constraint that continues to hamper leprosy research. As research animals, such as mice or armadillos, are the only reproducible method of sustained laboratory growth, this is restricted to a few specialized laboratories. The development of axenic media would increase access to this field.

**Methods:**

We performed a descriptive bibliographic review (May 2025) across PubMed, Embase, and the Cochrane Library using both controlled vocabulary and free terms related to *M. leprae* cultivation. After de-duplication and screening, 78 studies met the inclusion criteria.

**Results:**

Historically, claims of *in vitro* growth on egg-based or synthetic media have proven irreproducible or were subsequently attributed to non-leprae mycobacteria. Temperature and gas composition emerge as critical parameters: convergent evidence indicates thermosensitivity with optimal performance at 30–33 °C and deterioration at 37 °C; limited growth has been reported under microaerophilic atmospheres (~2.5% O₂ with added CO₂), yet durable subculture remains unachieved. Cell-based systems—such as macrophages or Schwann cells, which are traditional targets *in vivo* of *M. leprae*—can preserve viability for weeks and occasionally increase bacterial counts, but continuous, exponential replication has not been demonstrated. Mechanistic insights from genomics, transcriptomics, and metabolomics suggest that while *M. leprae* presents extensive gene decay, many core biosynthetic pathways persist. Lipid droplets (LD), immunomodulators (e.g., IL-10, PGE₂, and IGF-I), and L-arginine/nitric oxide pathways appear to shape the intracellular fate of the bacterium. An alternative unifying hypothesis posits that failure *in vitro* reflects structural fragility rather than a single auxotrophy, with host-derived factors (e.g., LDs and iron delivery) transiently compensating *in vivo*.

**Discussion:**

To date, no reproducible, cell-free culture system exists. This review aims to provide a starting point for future research into this objective. Achieving a reproducible *in vitro* culture of Hansen’s bacilli would represent a major advance in the field of leprology and would significantly accelerate translational research in this disease.

## 1 Introduction

*Mycobacterium leprae* is the causative agent of leprosy, together with the latterly described *Mycobacterium lepromatosis* (Singh et al., 2015). Leprosy is a chronic, yet curable disease affecting the skin, peripheral nerves, eyes and mucous membranes of the upper respiratory tract (Britton and Lockwood, 2004). The leprosy bacillus was the first human pathogen identified as causing disease in humans. It was identified by Hansen in 1874 from tissue derived from leprosy patients (Ghosh and Chaudhuri, 2015). *M. leprae* is a gram-positive, acid-fast bacillus (AFB) considered an obligate intracellular pathogen owing to its persistent inability to grow in axenic media over the past 150 years (Ojo et al., 2022).

There have been several claims of successful cultivation of *M. leprae*, both in axenic media and cell culture. Pattyn (1973) offered a compilation of authors reporting successful mycobacterial isolation, including Bapat (1989), Nojima, Mashelkar and Bhat, and Nadkarni and Coutinho on the Indian Cancer Research Centre (ICRC) bacillus strain C-44; Binford and Reich on the NQ strain; de Souza Araujo, Mabalay, Lu Huynh-Thanh, Nakayama, and Fauchet (references for these authors are not available). However, Pattyn emphasized that none of these isolates were examined by taxonomists familiar with non-tuberculosis mycobacteria, raising doubts about their authenticity.

In some instances, researchers have reported supposed mycobacteria strains with morphological divergence from the known varieties. Pattyn (1973) also referenced Martinova and Reich, who described *micrococcus*-like forms; Parès, who observed L forms; and Gay Prieto, who reported submicroscopic forms via electron microscopy (original articles, in which these varieties are described, are not available). Sanjuan even described a cycle of morphological variations in *M. leprae* occurring under *in vitro* conditions (Sanjuán Nadal, 1946).

Baker reported Rogers and Muir’s classification (the original report of the classification is not available) of bacilli isolated from lepromatous tissue, which divides them into four main morphological categories (Baker, 1983):

- Diphteroids or Streptothrix strains: pleomorphic bacilli that grew readily on conventional media and were labile in acid-fastness. Characteristically, they stained gram-negatively in early culture stages and developed acid-fastness with time (Dhople, 1987). These were reported by Delville and Pichel (1975) and Skinsnes et al. (1978).
- Chromogenic acid-fast bacilli. The group most frequently confused with *M. leprae*. Their biological characteristics are compatible with spontaneously occurring non-pathogenic mycobacteria. Primary culture is difficult to obtain, but afterwards, subcultures yield rapidly growing colonies of pleomorphic bacilli (Dhople, 1987). Skinsnes et al. (1978), on hyaluronic acid medium, and Kato and Ishaque (1977) on KI medium (also known as MacConkey agar) cultivated a strain of mycobacteria that follows this pattern.
- Non-chromogenic acid-fast bacilli. Slow moist-grown organisms requiring special media, they have constant growth features and resemble tinctorially the tubercle bacillus and morphologically the diphtheria bacillus (Dhople, 1987).
- Anaerobic bacilli. No reports are available describing these bacilli.

Although this classification is no longer applicable, we report it here to help understand the reports of previous authors who used it and to highlight the difficulty in interpreting the results published in the literature.

These conflicting reports regarding the *in vitro* isolation of the bacilli led to the establishment of different criteria to identify the isolated microorganism (Baker, 1983). Nevertheless, current advances in genomics permit easy typification of bacteria (Wheeler, 2003), which can be expected to expedite the identification process and rapidly confirm or reject new claims, allowing more agile research into the *in vitro* growth of *M. leprae*.

Some of the culture media reviewed in this manuscript correspond to those used in the isolation of these non-tuberculous mycobacteria. We have decided to include them here to provide the most comprehensive overview of historical and recent attempts to culture *M. leprae*.

Shepard developed the first reproducible cultivation of the leprosy bacilli in the mouse foot-pad (MFP) model, a milestone in the field (Shepard, 1960). The technique consisted of inoculating small numbers (<10^4^) of the bacteria into the hind foot-pad of immune-competent mice (Levy and Ji, 2006). This allowed the study of this microorganism, discovering its slow multiplication, with a doubling time of 12–14 days (Ojo et al., 2022; Miyamoto et al., 2016). However, this technique remains labour-intensive and costly, requiring large numbers of experimental animals; a challenge further compounded by increasingly stringent ethical regulations.

Nevertheless, the extracellular growth of *M. leprae* in axenic media may not be an impossible task, as there have been reports of extracellular microcolonies found in armadillos (Kirchheimer and Storrs, 1971), supposed to arise from intracellular colonies that, on dissolution of infected macrophages, are too large to be rephagocytosed and therefore continue to grow extracellularly (Baker, 1983).

Leprosy still affects more than 120 countries, with around 200,000 new cases reported every year (WHO, 2025). Approximately 3 million individuals around the world suffer from physical disabilities secondary to the damage to peripheral nerves and subsequent sensorimotor loss caused by leprosy (Ojo et al., 2022). Thus, leprosy is not a disease of the past; it is a current public health problem in many low- and middle-income countries. Furthermore, research into *M. leprae* has been hindered by its lack of available *in vitro* culture techniques. The aim of this narrative review is to summarize the previous research on the cultivation of *M. leprae* in order to provide a starting point for new projects that will attempt to break this Gordian knot of microbiology.

## 2 Results

Traditional culture media used for mycobacterial isolation can be classified into solid and liquid media. Solid media include egg-based media such as Löwenstein-Jensen—which also contains potato starch and glycerol, as well as malachite green to inhibit the growth of other organisms—and Middlebrook 7H10 with oleic acid, albumin, dextrose, and catalase (OADC). Conversely, liquid media include Middlebrook 7H9 with ADC—bovine albumin, dextrose, and catalase—enrichment and Dubos Tween albumin broths. Under these conditions, growth of many mycobacterial species typically occurs at 37 °C and 5% CO₂. However, neither *M. leprae* nor *M. lepromatosis* exhibit growth in these standard media (Wallace et al., 2021; Sharma et al., 2020). Although belonging to the same genus, different mycobacterial species require distinct growth conditions.

### 2.1 Cell-free media

Table 1 offers a summary of the composition of axenic culture media included in this review, with supplements and temperature of incubation, ordered by publication date. The number of times the culture media have been studied is indicated in Annex 1. The “Time” refers to the lag of time required to identify signs of supposed bacterial replication or the time after which bacillary metabolic activity waned. Empty boxes (−) correspond to categories for which no information was provided by the authors referenced.

**Table 1:** Composition of axenic culture media.

Medium	References	Composition	pH	Supplements	Incubation temperature (°C)	Time	Reported results
BME medium	Delville and Pichel (1975)	NaCl 6.8 g/L KCl 0.4 g/L MgSO4 0.1 g/L CaCl2.2H2O 0.2 g/L NaH2PO4. H2O 0.14 g/L NaHCO_3_ 2.2 g/L + L-arginine HCl 21 mg/L L-cysteine HCl 16 mg/L L-histidine 8 mg/L L-isoleucine 26 mg/L L-leucine 26 mg/L L-lysine HCl 36.47 mg/L L-methionine 7.5 mg/L L-phenylalanine 16.5 mg/L L-threonine 24 mg/L L-tryptophan 4 mg/L L-tyrosine 26 mg/L L-valine 23.5 mg/L +Dextrose 1 g/L Phenol red 10 mg/L +Biotin, choline chloride, folic acid, nicotinamide, calcium pantothenate, pyridoxal, thiamine, and i-inositol (at concentration of 1 mg/L). Riboflavin at 0.1 mg/L	-	10% foetal calf serum	30 or 36	12 weeks	Weak acid-fast bacilli of diphteroid characteristics (morphologic characterization) *Assume to be Corynecbacterium* spp.
RPMI-1640 medium		Ca(NO_3_)_2_.4H_2_O 100 mg/L MgSO_4_ 48.84 mg/L KCl 400 mg/L NaHCO_3_ 2 g/L NaCl 6 g/L Na_2_HPO_4_ 0.8 g/L + Glycine 10 mg/L L-arginine 200 mg/L L-asparagine 50 mg/L L-aspartic acid 20 mg/L L-cysteine HCl 65 mg/L L-glutamic acid 20 mg/L L-glutamine 300 mg/L L-histidine 15 mg/L L-hydroxyproline 20 mg/L L-isoleucine 50 mg/L L-leucine 50 mg/L L-lysine 40 mg/L L-methionine 15 mg/L L-phenylalanine 15 mg/L L-proline 20 mg/L L-serine 30 mg/L L-threonine 20 mg/L L-tryptophan 5 mg/L L-tyrosine 29 mg/L L-valine 20 mg/L + Biotin 0.2 mg/L, choline chloride 3 mg/L, D-calcium pantothenate 0.25 mg/L, Folic acid 1 mg/L, niacinamide 1 mg/L, para-aminobenzoic acid 1 mg/L, pyridoxine 1 mg/L, riboflavin 0.2 mg/L, thiamine 1 mg/L, vitamin B12 0.005 mg/L, inositol 35 mg/L + Dextrose 2 g/L, glutathione 1 mg/L, phenol red 5 mg/L	-	10% foetal calf serum	30 or 36	12 weeks	Weak acid-fast bacilli of diphteroid characteristics (morphologic characterization) *Assume to be Corynecbacterium spp*
Dubos medium	Delville and Pichel (1975)	0.5 g/L pancreatic digested casein, 2 g/L of asparagine, 0.2 g/L of polysorbate, 1 g/L of KH_2_PO_4_, 2.5 g/L of Na_2_HPO_4_, 50 mg/L ferric ammonium citrate, 10 mg/L of MgSO_4_, 0.5 mg/L CaCl_2_.2H_2_O 0.1 mg/L of ZnSO_4_, 0.1 mg/L of CuSO_4_	-	10% foetal calf serum	30 or 36	12 weeks	Acid-fast bacilli of diphteroid characteristics (morphologic characterization) *Assume to be Corynecbacterium spp*
	Dhople et al. (1988)		-	-	34	8 weeks	No multiplication Failure in MFP model
	Biswas (1997)		-	0.01% thyroxine sodium	37	8–16 weeks	Weak acid-fast bacilli (morphologic characterization and behaviour in MFP model)
KI-1 medium	Kato and Ishaque (1977)	Potential energy sources: 2 g of heat stable yeast extract (Difco), sodium succinate, compounds containing SH groups (L-cysteine, penicillamine, and thioglucose), oleic acid, Tween 80 + 30 g of glycerol, in 1 L of phosphate-buffered saline (PBS)	-	Penicillin G-sodium 200 IU	34	10–20 days	Non-acid-fast filamentous bacilli, recovering acid-fastness after oxidation with periodic acid (morphologic criteria)
LA-3 medium	Skinsnes et al. (1978)	81 ml of 0.006 M phosphate buffer (Na_2_HPO_4_-KH_2_PO_4_), 3 ml of glycerine, 100 mg of sodium salt hyaluronic acid, 6 g of bovine serum albumin, 16 ml of fresh yeast extract and 20,000 U of potassium penicillin G, suspended in 0.6 ml of sodium-citrate buffer	6.2	2% reagent grade agar or 0.5% agarose for plate use	37 or 25	6 weeks	Non-acid-fast “protoplast” and “spheroblast” (morphologic criteria)
	Dhople et al. (1988)			-	34	8 weeks	No multiplication Failure in MFP model
Modified Eagle’s medium	Oltizki and Godinger (1967)	NaCl 6.8 g/L KCl 0.4 g/L MgSO_4_ 0.1 g/L CaCl_2_.2H_2_O 0.275 g/L NaH_2_PO_4_. H_2_O 0.125 g/L Glucose 1.0 g/L L-glutamine 0.3 g/L L-tyrosine 0.75 g/L L-arginine HCl 0.09 g/L L-histidine HCl 0.42 g/L L-isoleucine 0.105 g/L L-leucine 0.105 g/L L-lysine HCl 0.15 g/L L-methionine 0.03 g/L L-phenylalanine 0.067 g/L L-threonine 0.1 g/L L-tryptophane 0.015 g/L L-valine 0.095 g/L L-serine 0.042 g/L Aminoacetic acid 0.03 g/L L-cysteine 0.05 g/L + Biotin, choline chloride, folic acid, nicotinamide, calcium pantothenate, pyridoxal, thiamine, and i-inositol (at concentration of 1 mg/L). Riboflavin at 0.1 mg/L	-	Human foreskin extracts +Mycobacterial extracts (E2) +Non-acid-fast organisms extracts	37	8 weeks	Maintenance of viability of *M. leprae* for 8 months Multiplication of *M. leprae* in 8 weeks (Morphologic criteria)
NM3 medium		-	-	Glucoronic acid (0.2–1.0%) or Galacturonic acid (0.2–1.0%) or Citric acid (0.5–2%) or Pyruvic acid (0.5–2%)	-	-	Observed *in vitro* multiplication Inhibition by gluconic acid (No information on criteria used)
MY 14b agar medium	Nakamura et al. (1982)	KH_2_PO_4_ (4.0 g), CaCl_2_ (0.0025 g), asparagine (3.0 g), sodium pantothenate (0.1 g), Na_2_HPO_4_·12H_2_O (3.0 g), MgSO_4_·7H_2_O (0.1 g), sodium pyruvate (2.0 g), sodium citrate (2.0 g), sodium glutamate (3.0 g), glucose (10.0 g), Tween 80 (0.1 g), yeast RNA (100 μg), bovine serum albumin (BSA) Fraction V (Armour; 5.0 g), powdered agar (20.0 g) and water (1,000 ml)	6.6–6.8	-	37	7 weeks	No growth detected (Morphological criteria)
	Dhople et al. (1988)			-	34, 36	3 weeks	No multiplication Failure in MFP model
Nutrient-Tween medium	Lee and Colston (1985)	Difco Bacto nutrient broth 8 g + Tween-80 0.6 ml	-	Hank’s balanced salt solution +Tween-80 0.05%	37, 33, 4	2 weeks	Decrease in intracellular ATP (ATP assay criteria)
Thioglycollate medium		TG Difco	-				Decrease in intracellular ATP (*more rapid decrease*) (ATP assay criteria)
DH medium	Dhople et al. (1988)	Nakamura’s medium for *M. lepraemurium* Modifications: Malic acid (0.2%) replacing α-ketoglutaric acid *δ*-aminolevulinic acid (0.015%) replacing haemin,	7.0	Dithiothreitol and trace amounts of Fe^3+^, Zn^2+^, Ca^2+^, Co^2+^ and Mn^2+^	34	4 weeks	400–600% increase in cell mass up to 16 weeks, followed by rapid deterioration Failure of reinoculated bacilli in fresh media (Criteria used: ATP assay, DNA assay, Thymidine uptake, and Cell characterization)
Mahadevan’s medium		Eagle’s minimal essential medium (70%), foetal bovine serum (20%), and chick embryo extract (10%) Incubated with dorsal root ganglia for 3 days	7.2	20% human AB serum	34	4 weeks	
Middlebrook 7H9 medium		0.5 g/L of (NH_4_)_2_SO_4_, 0.5 g/L of L-glutamic acid, 0.1 g/L of sodium citrate, 1 mg/L of pyridoxine, 0.5 mg/L of biotin, 2.5 g/L of Na_2_HPO_4_, 1 g/L of KH_2_PO_4_, 0.04 g/L of ferric ammonium citrate, 0.05 g/L MgSO_4_, 0.5 mg/L of CaCl_2_.2H_2_O, 1 mg/L of ZnSO_4_, 1 mg/L of CuSO_4_	-	-	34	8 weeks	No multiplication Failure in MFP model
Wheeler’s medium	Wheeler (1988)	50 mM HEPES (N-2-hydroxyethylpiperazine-N′-2-ethanesulfonic acid), 5 mM NaCl, 1 mM MgSO_4_, 0.2 mM NaH_2_PO_4_, 17 mM asparagine, 55 mM glucose	7.0	6 M KOH +10 μM uracil, 10 μM thymidine and 10 μM cytosine, +Penicillin 50 IU/ml 1 μCi [G-^3^H] hypoxanthine was added to measure mycobacterial viability	34	15 days	No differences of incorporation of radiolabelled hypoxanthine in live and heat-killed bacilli (Criteria: rate of incorporated radioactivity)
Ishaque’s liquid medium	Ishaque (1990)	(NH_4_)_2_SO_4_ 0.2 g, KH_2_PO_4_ 2.0 g, glycerol 2.5 g, MgSO_4_ · 7H_2_O 0.2 g, sodium thioglycolate 0.8 g, haemin 0.002 g, and 100 ml water	7.0	-	34	6–8 weeks	Lag period of 6–8 weeks, followed by rapid growth until 24 weeks. Then progressive decline (Criteria used: counting of acid-fast bacilli, ATP determination and DNA determination)
Ishaque’s solid medium			-	200 ml of egg yolk for every 100 ml of liquid medium			
ML medium	Osawa (1997b)	Horse serum 5 ml Glucose 15 mg Waymouth medium powder without glutamine 353 mg L-glutamine 30 mg L-cysteine 30 mg Nicotinamide adenine dinucleotide 6 mg Ascorbic acid 6 mg Cyclodextrin 30 mg Yeast extract (Difco) 30 mg Cytochrome C (Boehringer) 1 mg Glycerol 0.25 ml Glucuronic acid 30 mg N-acetyl-glucosamine 30 mg Hemin 2 g (in 0.5 ml of 1 N NaOH) Final volume 30 ml	6.5	Filter sterilized (0.2 μm) Some experiments had addition of Cutina LE (mixture of glyceryl stearate and sodium stearylsulfate) in cylinders 4 mm in diameter and 2–10 mm in length 20 μg/ml of streptomycin and 40 U/ml of penicillin G	32	5 weeks (1 weeks with cutina LE)	Growth in 75 days (addition of Cutina LE increased growth to 1 week) (Criteria used: increased optical density and quantitative PCR)
NHDP medium	Ojo et al. (2022)	7H9 powder 4.68 g/L, bovine albumin (Fraction V) 5.0 g/L, dextrose 8.0 g/L and casitone/casein hydrolysate 1.0 g/L	-	-	33	48 and 96 h	Maintenance of metabolic activity for up to 96 h (Criteria used: radiorespirometry and viability staining)
NK-180 medium	Nakamura and Matsuoka (1998)	ACES (N-(2-acetamido)-2-aminoethanesulfonic acid) buffer (0.4%), sodium citrate (0.1%), magnesium sulphate (0.06%), sodium glutamate (0.5%), dextran (1.0%), glycerine (2.0%), adenosine (15 μg/ml), egg lecithin (5 μg/ml), bovine serum (10%), egg yolk extract (10%), vitamin K3 (0.5 μg/ml), N-acetylglucosamine (0.02%) and 50 μg/ml of folinic acid	7.0	-	30	12 weeks	Increase of bacterial density by 12 weeks Increase of DNA by quantitative PCR Decreased of ATP
Kirchner medium	Nakamura and Matsuoka (2001)	Na_2_HPO_4_.12H_2_O 19 g, KH_2_PO_4_ 2 g, MgSO_4_.7H_2_O 0.6 g, Na_3_C_6_H_5_O_7_.2H_2_O 2.5 g, L-asparagine 5 g, Glycerol 20 ml, 0.4% phenol red 3 ml + Distilled water to 1 L	7.0	Adenosine (50 μg/ml), thymidine (100 μg/ml), pyruvic acid (0.1%), transferrin (10 μg/ml), folic acid (10 μg/ml), egg yolk extract (10%) and bovine serum (10%)	30	6 weeks	Increase of DNA and ATP content up to 4–6 weeks, followed by rapid decrease No significant increase in bacilli numbers by microscopy (Criteria used: Bacillary counting, ATP extraction and quantitative PCR)
NK-260 medium	Nakamura (2001)	KH_2_PO_4_ (0.3%), Na_2_HPO_4_ (0.6%), sodium citrate (0.25%), MgSO_4_ (0.06%), glutamate (0.8%), glycerine (2%), and adenosine (50 μg/ml)	7	5 N KOH +foetal calf serum (10%), egg yolk extract (10%), pyruvate (0.2%), and transferrin (10 μg/ml)	30	6 weeks	ATP and DNA increase up to 4–6 weeks. No significant increase in bacillary numbers (Criteria used: Bacillary counting, ATP extraction and quantitative PCR)
	Amako et al. (2016)			+ 10–20% human blood plasma or nude mouse tissues grounded in phosphate-buffered saline (PBS)	30–32	60 days	Non-exponential growth in supplemented media (30–40% non-viable) (Criteria used: bacillary count, viability staining, quantitative PCR)

#### 2.1.1 BME, RPMI, and Dubos media

In 1975, Delville and Pichel incubated biopsies from leprosy patients in different liquid media: BME (Basal Medium Eagle), RPMI (Roswell Park Memorial Institute), and Dubos media, supplemented with 10% foetal calf serum (FCS; Delville and Pichel, 1975). The authors also cultivated peripheral blood from these patients in Dubos liquid medium, without Tween, and in the same medium supplemented with casitone, yeastolate, pyruvate, and glycerine (Delville and Pichel, 1975).

The authors observed colony formation after 1–2 weeks of culture in all prior media. Subculturing of isolated bacilli in Löwenstein–Jensen solid medium without malachite green, Dubos liquid medium, or solid medium enriched with 10% calf or horse serum, sometimes with casitone, yeastolate, and pyruvate, achieved growth even more rapidly at either 30 or 36 °C (Delville and Pichel, 1975).

Isolated bacilli were morphologically characterized, being described as “diphtheroids” (Delville and Pichel, 1975), with poor affinity for Ziehl–Neelsen staining. Based on their observations, the coexistence of non-acid-fast bacilli with acid-fast bacilli (AFB) observed in leprosy lesions histological studies suggested that *M. leprae* may present two stages of evolution. Non-AFB were thought to be present before AFB and to have a higher membrane permeability to allow easier metabolic exchanges to enhance their growth and reduce generation time (Delville and Pichel, 1975). Due to their behaviour *in vitro*, these “diphtheroid” isolates are compatible with *M. scrofulaceum*.

Dhople et al. (1988) experimented on Dubos medium with bacteria incubated at 34 °C but reported no evidence of *M. leprae* multiplication or viability.

Twenty years later, Biswas SK supplemented Dubos synthetic medium with 0.01% thyroxine sodium, a stimulant of somatic cell metabolism and protein synthesis, and modified the incubation temperature to 37 °C. After a difficult primary culture, subcultures showed large numbers of bacilli within 2–3 weeks of inoculation. The weak acid-fastness observed in the microorganisms was attributed by the author to a lipid coat depleting effect of thyroxine. When the cultured bacteria were injected into MFP, the animals developed a red and swollen lesion within 30–35 days post-inoculation (Biswas, 1997).

In this same article, the author claimed that bovine serum albumin (BSA) and egg albumin could depress the growth of *M. leprae* (Biswas, 1997).

#### 2.1.2 KI-1 medium

Kato and Ishaque prepared culture media based on the KI-1 (Kato-Ishaque) medium. In this basal medium, known oxidizable substrates of *M. leprae* (included in Table 1) were incorporated as potential energy sources. Additional carbon and nitrogen sources were also added (Kato and Ishaque, 1977).

The progress of the cultures was assessed by the appearance of sediment or turbidity in the liquid media, and when bacillary counts per field “left no doubt that the number of bacilli (…) increased at least twice or four times relative to baseline” (Kato and Ishaque, 1977). Under these conditions, reported growth was initially slow and depended on inoculum size and source. However, once incipient growth was observed within 10 to 20 days in the primary culture, a dense bacillary precipitate usually developed within the following 2 weeks. Secondary inoculation into the homologue medium resulted in faster growth compared to the primary culture (Kato and Ishaque, 1977).

Bacilli isolated from subcultures were able to grow on Löwenstein–Jensen (solid medium) and Dubos (liquid medium) media, which suggested to the authors a possible adaptation to extracellular life. However, the reports concluded that the *in vitro* grown bacilli obtained might be morphologically compatible with *M. scrofulaceum* species. No genomic identification of the isolate was reported in the article (Kato and Ishaque, 1977).

#### 2.1.3 LA-3 medium

Skinsnes et al. (1975) developed the Leprosy Atelier (LA)-3 medium after observing the effect of supplementation with 0.25 g of hyaluronic acid (HA) in 10 ml of PBS administered intraperitoneally weekly in *M. leprae*-infected nine-banded armadillos (Skinsnes et al., 1978; Skinsnes et al., 1975).

HA administration appeared to have an infection enhancement effect in the experimental animals, as the authors reported a 100% infection rate, with disseminated mycobacteriosis and a four-fold increase in the number of bacilli recovered. Compared with reported infection rates of 30–40%, these results represented a significant increase, although the study suffered from the limitation of a small sample size, with only six experimental animals used (Skinsnes et al., 1978).

Nevertheless, the authors concluded that HA facilitated extracellular mycobacterial existence through the enablement of a more efficient cell wall repair mechanism, permitting bacilli to maintain their viability and replicate more rapidly (Skinsnes et al., 1978).

Skinsnes et al. (1975) performed 23 experiments using LA-3 medium and observed growth in 7 of them, 2 of which died out after 2 subcultures. As in the reports of Kato and Ishaque (1977) and Delville and Pichel (1975), initial isolates were difficult to obtain and only demonstrated growth in LA-3 or KI-1 media after a long period of adaptation during which the AFB disappeared and were substituted by amorphous non-acid-fast materials, which were named by the authors as “protoplast” and “spheroblast” according to their morphology. These forms were thought to be part of the ongoing mycobacterial reproductive process (Skinsnes et al., 1975). As in the previous reports, presumed leprosy isolates showed similar characteristics to the *M. scrofulaceum* species (Skinsnes et al., 1978).

#### 2.1.4 Eagle’s medium and NM3 medium

Oltizki and Godinger (1967) reported the successful cultivation of *M. leprae* on a modified Eagle’s medium containing human foreskin extracts and mycobacterial extracts (obtained by ultrasonic vibration of culturable mycobacteria followed by filtration through Seitz filters), as well as extracts from non-acid-fast organisms (such as yeasts and *Escherichia coli*; Oltizki, 1977).

Years later, Oltizki used medium NM3, supplemented with glucuronic or galacturonic acid (0.2–1.0%) and citric and pyruvic acid (0.5–2%), and reported enhanced *in vitro* multiplication of *M. leprae*, whereas gluconic acid, at a concentration of at least 0.02%, inhibited growth (Oltizki, 1977). No further information on this medium or the experimental conditions is available, as only a reference in Oltizki’s (1977) study survives and access to the original manuscript is now unavailable. Thus, Oltizki reported apparent growth in both axenic media, although the composition of the medium appeared to influence the mycobacterial growth rate.

#### 2.1.5 MY 14b agar medium

Nakamura et al. evaluated leprosy bacilli growth on Murohashi–Yoshida (MY) 14b agar medium (Nakamura et al., 1982). No colony of *M. leprae* was obtained under these conditions (only small yellow-white spots corresponding to tissue debris), and no analysis evidenced an increase in the number of bacterial cells. The viability of leprosy bacilli was reported to be completely lost within 7 weeks of cultivation in the medium (Nakamura et al., 1982).

#### 2.1.6 NT and TG medium

Lee and Colston studied the metabolism (measured by ATP levels) of *M. leprae* suspended in Hank’s balanced salt solution (HBSS) containing 0.05% Tween-80, but without phenol red; in nutrient-Tween medium (NT) or in thioglycollate medium (TG; Lee and Colston, 1985). They observed that ATP content exponentially declined when stored over a 2-week period at 4 °C. Different storage temperatures modified the decay rate (see later in the Temperature section).

In addition, treatment with 0.5 mM NaOH for 1 h at room temperature (a common technique to remove contaminants) did not produce a significant decrease in ATP levels (Lee and Colston, 1985).

The authors also observed that *M. leprae* was unable to incorporate ATP from its surrounding environment, making *M. leprae*’s reliance on host ATP for its survival unlikely. Instead, bacilli appeared capable of generating ATP *de novo*, even during *in vitro* incubation, as evidenced by the authors through the study of the incorporation of ^32^P into ATP after 24 h *in vitro* (Lee and Colston, 1985).

#### 2.1.7 DH, Mahadevan’s conditioned medium, MY, LA-3, Dubos, and Middlebrook 7H9 media

Dhople et al. studied the growth of *M. leprae* in six different media, including: Dhople–Hanks (DH) medium (as a modification of Nakamura’s medium for *M. lepraemurium* incubated with 40% free air space in the culture tubes), Mahadevan’s conditioned medium, Murohashi–Yoshida (MY; Nakamura et al., 1982), LA-3 (Skinsnes et al., 1975), Dubos (Delville and Pichel, 1975; Dhople et al., 1988; Biswas, 1997), and Middlebrook 7H9 media (Dhople et al., 1988). All cultures were incubated at 34 °C (Dhople et al., 1988).

No sign of multiplication or viability of *M. leprae* was observed in either MY, SK, 7H9 nor Dubos media (Dhople et al., 1988). However, *M. leprae* retained its original metabolic activity and pathogenicity for up to 20 days in MY agar medium at 36 °C, indicating that MY could serve as a suitable medium for bacilli transportation (Dhople, 1987). In both DH and Mahadevan media, there was a lag period of 4 weeks followed by a 400–600% increase in cell mass at the end of 16 weeks of incubation, after which cells started to deteriorate. Subcultures of these bacilli in the homologous media under identical conditions showed a rapid decline of ATP levels over 12 weeks to baseline. The authors noted that a carry-over effect of host-derived components could not be excluded; thus, once the influence of these factors ceases, bacilli would rapidly degrade (Dhople et al., 1988).

#### 2.1.8 Wheeler’s medium

Wheeler developed a culture medium for the incubation of *M. leprae* at 34 °C for 24 h, followed by the addition of 1 μCi [G-^3^H] hypoxanthine in a final volume of 300 μl, with the aim of evaluating the uptake of hypoxanthine in purified suspensions of *M. leprae* as an indirect measure of bacterial viability (Wheeler, 1988).

The author reported no differences in radioactivity between bacteria from fresh tissue and from tissue stored at −70 °C for up to 11 months; hypoxanthine was also incorporated into suspensions of heat-killed bacilli, although at a lower rate than with live bacteria, with occasional individual experiments in which heat-killed bacteria incorporated radioactivity at a higher rate than some live bacteria. These results led Wheeler to conclude that hypoxanthine incorporation was not directly correlated with bacterial viability (Wheeler, 1988).

#### 2.1.9 Ishaque’s media

Ishaque developed two media for *in vitro* cultivation of *M. leprae*: a liquid medium and a solid medium with the same composition, with the addition of 200 ml of egg yolk for every 100 ml of liquid medium (when agar was used, no multiplication occurred; Ishaque, 1990).

Ishaque proposed that microaerophilic conditions were required for the growth of *M. leprae*, suggesting that the preference of leprosy bacilli to grow in subcutaneous tissues was probably better explained by the estimated oxygen tension of 2.5% rather than by lower temperature. To test this hypothesis, Ishaque cultivated AFB in the media previously described, flushing each culture jar with a gas mixture of known composition and incubating them at 34 °C (Ishaque, 1990; Stevens, 1979).

Under these conditions, visible growth appeared after a lag period of 6–8 weeks, and a two-fold increase was observed after 12 weeks, except in the tubes incubated under 1% oxygen of atmospheric air, which showed no change in bacillary count. After the first 12-week period, growth plateaued in the tubes incubated under 5% O_2_ and 5–10% CO_2_, while it continued to increase by a four-fold increase after 18 weeks in tubes incubated under a 2.5% O_2_ and 5 or 10% CO_2_. This was followed by a stabilization phase up to 24 weeks, after which there was a gradual decline until AFB disappearance at 42 weeks. Thus, Ishaque concluded that 2.5% O_2_ concentration was optimal compared to 5 or 1%, or atmospheric air; and a gas mixture containing 10% CO_2_ was superior to 5% (Ishaque, 1990).

When evaluating ATP and DNA concentration in the isolated bacteria, the author reported a two-fold multiplication of *M. leprae* in the solid medium compared to the liquid medium, followed by a decline in bacilli numbers after the third or fourth subculture, with no bacillus detected after the fifth transfer. Thus, it was concluded that “the problem of *in vitro* cultivation of *M. leprae* cannot be solved merely by providing the right gaseous environment” (Ishaque, 1990).

#### 2.1.10 ML medium

Osawa (1997a) developed the ML (*Mycobacterium leprae*) medium after reports of successful *in vitro* culture of *M. leprae* in murine macrophages, attempting to use it in axenic culture (Osawa, 1997b). When bacilli were cultured in 0.9 ml of ML medium at 32 °C in a glass culture flask, the medium became progressively turbid after 1 month, with optical density doubling in 65 days. In some cultures, the authors added 15 cylinders of Cutina LE (a mixture of glyceryl stearate and sodium stearyl sulphate), which resulted in an apparently higher growth rate of *M. leprae*, with a bacterial increase of over 10^3^-fold for 4 months. Continued re-inoculation was successful 18 times, with maintenance of the cultured strain for over a year. The identity of the isolated AFB was reported to have been confirmed with PCR (Osawa, 1997b).

#### 2.1.11 NHDP medium

Ojo et al. incubated harvested bacilli from the mouse foot pad (MFP) in NHDP (National Hansen’s Disease Program) medium at 33 °C (Ojo et al., 2022), and their viability was analysed at 48 and 96 h using radiorespirometry (Franzblau et al., 1992) and viability staining (BacLight Viability Staining Kit, Life Technologies). No significant differences in viability between freshly harvested *M. leprae* and those bacilli held in axenic medium for 48 and 96 h were observed. When gene expression profiles were analysed, a significant alteration in them was found, which increased with the time the bacilli were maintained in axenic media (Ojo et al., 2022).

While the majority of genes related to glucose metabolism maintained their expression, 76% of genes involved in lipid metabolism were downregulated (ascribed to the lipid-poor environment). Dihydropteroate synthase (*folP1*) and dihydrofolate reductase (*folA*) expression were reduced, all ATP synthase subunit genes were downregulated, and no significant changes in amino acid biosynthesis were detected. Genes associated with the cell envelope were also significantly increased, which was interpreted as an effort to maintain bacterial cell wall integrity (Ojo et al., 2022).

#### 2.1.12 Systematic approach of Nakamura M to *in vitro* cultivation of *M. leprae*

Nakamura evaluated several culture conditions in an attempt to identify the best combination of them which would enable *in vitro* growth of the fastidious bacilli.

- After identifying adjustment of the medium pH to 6.0 as the key to *in vitro* cultivation of *M. lepraemurium*, he evaluated its effect on the *in vitro* growth of *M. leprae*. Using phosphate buffers (KH_2_PO_4_:Na_2_HPO_4_; 0.05 M) at different pH levels as basal culture media, with the addition of 10% FCS, in tubes containing 5.5 ml of inoculated medium and 27.7% of free air space and incubated at 30 °C, Nakamura reported that, while ATP decreased at pH 5.5, 6.0, and 6.6, there was a 30–50 and 10% increase from baseline at pH 7.0 and 7.5, respectively, after 4 weeks of incubation. This increase was not observed when the incubation temperature was 37 °C, and if FCS was omitted, there was a rapid loss of bacterial activity after 2 weeks of incubation (Nakamura, 1995a).
- Glycerine concentration of 2% in culture medium was found to be optimum (with an increase of 159.3% in ATP after 4 weeks), compared to the toxic effect of 5 and 10%. In addition, dextran of a molecular weight ranging from 200,000 to 300,000 was more effective than that from 100,000 to 200,000, and in a concentration of 1% was superior to 0.5%. Nakamura reported that the addition of glycerine 2% at pH 7.0 maintained bacilli viability for 16 weeks, albeit at progressively lower levels (Nakamura, 1996).
- The effect of the inoculum size (measured in ATP pg) on the metabolic activity of inoculated bacteria was evaluated through the study of bacterial suspensions of known concentration in PBS (pH 7.0) with bovine serum 10% or in PBS with 2% glycerine, incubated at 30 °C. Inocula of at least 3,000 pg. of ATP were reported to maintain their activity for 4 weeks, in contrast to an inoculum of smaller size (Nakamura, 1995b). These observations are also supported by other authors, although a carry-over of host factors into the primary culture to promote initial growth cannot be excluded to explain this finding (Baker, 1983).

Based on these results, Nakamura and Matsuoka designed a culture medium (NK-180), in which silicone-coated slides were submerged and incubated at 30 °C and pH 7.0. There was a two-fold increase in bacterial numbers after 12 weeks, with a two to four-fold increase in DNA, but with a linear decrease in ATP content. This suggested to the authors that bacteria were using energy reserves acquired previously *in vivo*. No growth took place when non-silicone-coated slides were used (Nakamura and Matsuoka, 1998).

After the previous experience, Nakamura designed the culture medium Nakamura–Kirchner (NK)-260, on the basis of Kirchner medium. The author reported that incubation of *M. leprae* in this medium allowed a significant increase in intracellular ATP content in *M. leprae*, which peaked at 4–6 weeks after inoculation. This increase was greater when adenosine was added to the medium, with an optimal concentration of 50 μg/ml. However, no significant increase in bacterial cell numbers was reported, and metabolic activity was not maintained longer than 6 weeks.

When transference to a new medium was attempted after 6 weeks, through a centrifugation at 1,500 × *g* for 20 min, it resulted in the loss of 74% of ATP content (Nakamura and Matsuoka, 2001). This loss was ascribed to stress suffered by bacterial cells during the transference process (Nakamura, 2001). The addition of catalase did not improve ATP production, and attempts to reinforce the cell wall were unsuccessful (sucrose proved toxic; polyvinylpyrrolidone showed a non-significant improvement; glucuronic acid or N-acetylglucosamine had no effect; and glycine was able to maintain ATP production for up to 10 weeks). With this precedent, Nakamura suggested that fixation of *M. leprae* to glass slides improved results compared to liquid suspensions.

Using the NK-260 medium, Amako et al., achieved only a slight increase in the number of leprosy bacilli after the addition of 10–20% human blood plasma or nude mouse tissues homogenized in phosphate-buffered saline (PBS) and passed through a 0.2-μm filter. The growth rate was slow, taking more than 60 days, and non-exponential; acid-fast bacilli (AFB) were isolated in late-stage cultures aggregating in large masses in which 30–40% of bacilli showed signs of non-viability. The authors proposed the theory that degenerated bacteria in globi might release nutrients or growth factors which may support the growth of the remaining bacteria (Amako et al., 2016).

#### 2.1.13 Temperature effect on *M. leprae* cultivation

The optimal incubation temperature of *M. leprae* culture has been a point of contention.

In 1985, Lee and Colson observed that different storage temperatures modified the decay rate of ATP content in a preserved bacillary suspension. The half-life was 10 h at 37 °C, 21 h at 33 °C and 2.5 days at 4 °C (Lee and Colston, 1985). Furthermore, freezing and thawing resulted in a 30–40% decrease in ATP content when bacilli were suspended in HBSS medium, and a 20% decrease when in TG medium. Storage of bacilli at 4 °C led to an exponential decline in intracellular ATP content over a two-week period, with similar decay rates between NT and HBSS media (half-lives of 2.7 and 2.5 days, respectively), and slower in TG (half-life of 4.5 days).

In 1988, Dhople et al. analysed oxygen uptake by *M. leprae* in axenic media incubated at different temperatures (Dhople et al., 1988). It was reported that the oxygen consumption was higher at 34 °C than at 30, 32, and 37 °C.

Using radiorespirometric analysis, Tuman and Krahenbul measured the metabolic activity of leprosy bacilli maintained in 4 ml of Middlebrook 7H12 medium incubated at −80, 4, 25, 33, and 37 °C for up to 3 weeks, reporting that the optimal temperature for the growth of *M. leprae* was 33 °C (Truman and Krahenbuhl, 2001). Samples maintained at −80 °C lost almost all viability after only thawing once, while incubation at 37 °C for only a few days had extremely deleterious effects.

Wheeler reported no differences in 1 μCi [G-^3^H] hypoxanthine uptake by *M. leprae* incubated in Wheeler’s medium between bacteria from fresh tissue and from tissue stored at −70 °C for up to 11 months. Furthermore, the author observed that although storage of bacilli at 4 °C resulted in a decrease in viability (measured by radioactivity) exponentially with time, hypoxanthine incorporation increased for the first 15 days before decreasing. Thus, no conclusion could be reached on the effect of temperature storage, as hypoxanthine uptake was not a reliable indicator of bacterial viability (Wheeler, 1988).

The effect of storage temperature was also studied by Kohsaka et al., where the authors evaluated the effects of lyophilization in different media (sterile distilled water, water containing 10% FCS or 10% skim milk-water) on *M. leprae* viability. The lyophilization process involved the freezing of bacterial suspension at −60 °C with dry ice-alcohol, then lyophilization for 4–16 h and, finally, ampoule storage at 4 °C. Sample reconstitution was achieved through the addition of 1 ml of distilled water to the remnant, and then a 10-fold dilution in PBS. The authors reported a 10^−2^- to 10^−3^-fold reduction in viable AFB from the original sample. The best results were obtained with the skim milk–water solution, with viability levels after 2 years’ storage at 4 °C similar to the initial sample. Additionally, a slow freezing procedure (<1 °C/min), rather than a quick freezing with liquid nitrogen, also improved viability. The loss of viable bacilli was thought to be due to bacilli fragility (Kohsaka et al., 1993).

However, the most solid evidence available for the optimal culture temperature of the leprosy bacilli was reported by Kim et al. (2019). As no *M. leprae in vitro* growth had been achieved at that point, the authors developed a *Mycobacterium smegmatis* that usually grows between 30 to 45 °C, with its DNA gyrase genes (*gyrB* and *gyrA*) replaced with *M. leprae* homologue genes (Kim et al., 2019). After this modification, no colonies were achieved on 7H10 agar plates or 7H9 liquid medium when they were incubated at 37 °C; whereas cells grew uniformly at 30 °C and 33 °C.

In addition, the authors further characterized the enzymatic activity of *M. leprae* DNA gyrases, reporting a 50% loss of activity at 37 °C compared to 30 °C (in comparison, *M. tuberculosis* DNA gyrase maintained its activity up to 42 °C). Gyrases could be implicated in the determination of some of these conditions due to their relevance for DNA replication, transcription, and recombination. Consequently, environmental conditions that hampered the activity of gyrases could render DNA replication impossible and, thereby, limit bacterial proliferation. Thus, it was concluded that the optimum growth temperature for *M. leprae* was 30 °C, conditioned by its thermosensitive DNA gyrase (Kim et al., 2019).

### 2.2 Cell cultures

Table 2 presents the composition of cell culture media included in this review, along with incubation temperature. Empty boxes (−) correspond to categories for which no information was provided by the authors referenced.

**Table 2:** Composition of cell culture media.

Cell culture	References	Culture media	Supplements	Incubation temperature (°C)	Reported results
Macrophages	Mouse-derived macrophages				
	Chang and Neikirk (1965)	Medium 4:5:1 40% horse serum 50% NCTC109 medium 10% of a dilution of 1:5 beef embryo extract in NCTC medium	liver extract L fraction (1 mg/ml) and ferric nitrate (2 μg/ml)	30	No signs of growth (Morphological criteria)
	Matsuo and Utsunomiya (1976)	Eagle’s minimum essential medium	10% foetal calf serum (FCS)	25 31 33 37	No signs of growth (Morphological criteria and MFP)
		L-15 medium	2% FCS	30	No signs of growth (Morphological criteria and MFP)
	Sharp and Banerjee (1984)	NCTC 109 medium + Bone marrow cell culture medium	40% horse serum or FCS 2% beef embryo extract ampicillin (100 μg/ml) 10 IU/ml of preservative-free heparin	35 or 30	Increase in counts of acid-fast bacilli (probably due to break up of globi or fragmentation of bacilli) (Morphological criteria)
	Osawa (1997a)	Macrophage culture: 20% heat-inactivated horse and 80% Waymouth solution Infection medium: Fresh medium Incubation medium: 20% heat-inactivated horse serum (16 ml) Waymouth solution (58 ml), glucose–glutamate solution (1.2 ml; glucose 3 g and L-glutamate 102.3 mg in 10 ml of 0.85% NaCl), L-cysteine (80 mg), nicotinamide adenine dinucleotide (16 mg), ascorbic acid (8 mg), cyclodextrin (80 mg), hemin (80 mg), yeast extract (80 mg)	100 IU/mL of penicillin 100 μg/ml of streptomycin NaHCO_3_ to adjust pH to 7.0 5% normal mouse serum	37	Significant multiplication of *M. leprae* with extracellular coccal shape (Criteria used: PCR identification at beginning, morphological criteria)
	Human-derived macrophages				
	Drutz and Cline (1972)	McCoy’s medium	30% pooled AB normal human serum	37, 34 or 31	Incorporation of tritiated thymidine in intracellular bacilli (not enough follow-up to determine growth or maintenance of viability)

	Samuel et al. (1973)	5 ml of Eagle medium	2.64 g/L of NaHCO_3_ 40% autologous serum 100 IU/ml of penicillin	37 or 33	2- to 9-fold increase in bacilli numbers (Morphological criteria)
	Sibley and Krahenbuhl (1988a)	RPMI 1640 medium	1 mM glutamine, 20 mM HEPES (N-2-hydroxyethylpiperazine-N′-2-ethanesulfonic acid) buffer, 100 U/ml penicillin, and 20% heat-inactivated foetal bovine serum (FBS)	-	Increased levels of PGE2 in macrophages infected with viable *M. leprae* during the first 7 days Activation by IFN-gamma was inhibited in heavily infected macrophages
	Hagge et al. (2004)	DMEM containing, HEPES and sodium bicarbonate, 10% FBS, 2 mM L-glutamine, and 50 μg/ml gentamicin	5 μg/ml monocyte-colony-stimulating factor (M-CSF)	37, prior to *M. leprae* infection 33, after infection	No information on maintenance of metabolic activity or growth on culture
	Adams et al. (1991)	Phenol red-free DMEM + 0.4 mM L-arginine	20 mM glucose, 20 mM HEPES, 2.5 g/L NaHCO_3_, 2 mM L-glutamine, 100 μg/ml ampicillin and 10% heat-inactivated FCS	-	No information on maintenance of metabolic activity or growth on culture
	Moura et al. (2007)	RPMI 1640 medium	2 mM L-glutamine, 100 U/ml penicillin, 100 μg/ml streptomycin and 10% heat-inactivated FCS	37	Decline of infected macrophages from 35% on day 1 to 4% on day 24 (Morphological criteria)
	Fukutomi et al. (2004)	RPMI 1640 medium L-arginine-free	20 mM glucose, 20 mM HEPES, 2.5 g/L NaHCO_3_, 2 mM L-glutamine, 100 μg/ml ampicillin and 10% heat-inactivated FCS	-	No differences in metabolic activity at day 9 of *M. leprae* from macrophages co-cultured with and without PMBC (Criteria used: oxidation rate of ^14^C-palmitic acid)
Batista-Silva et al. (2016)	RPMI 1640 medium containing 2 mM L-glutamine	10% FCS, penicillin and streptomycin	37	No information on maintenance of metabolic activity or growth on culture of *M. leprae* (Data on viability refer to *M. smegmatis*)
Schwann cells	Einheber et al. (1993)	Minimal Essential Medium	10% FBS, 2 mM glutamine, 0.4% glucose and 50 ng/ml 2.5S nerve growth factor (NGF)	37	No information on maintenance of metabolic activity or growth on culture of *M. leprae*
	Mukherjee and Antia (1985)	70% Dulbecco’s modified Eagle medium, containing glucose (6 mg/ml), FCS (20%), chicken embryo extract (10%) and penicillin (100 U/ml)	-	37	10 to 12-fold increase of AFB in Schwann cells (progressive formation of globi), peaked on day 28 and declined afterward (Criteria used: morphology; tritriated thymidine uptake by *M. leprae*; growth on common media)
Complex tissue	De Paula et al., (2024)	DMEM	10% FBS and 1% antibiotic solution (100 U/ml penicillin and 100 mg/ml streptomycin)	37	Amplification of 16S rRNA of *M. leprae* at day 60 (Criteria used: quantitative PCR, MFP model)
Tick cell lines	Ferreira et al. (2018)	L-15B (81)	10% FBS, 10% tryptose phosphate broth (TPB), 0.1% bovine lipoprotein–cholesterol concentrate and 2 mM L-glutamine	30	Increase in the number of bacilli over a 20 day period in the IDE8 cell line (Criteria used: morphology, 16S rRNA/16S rDNA ratio by qPCR and MFP model)

In 1987, the Committee on Bacteriology and Pathology, on the occasion of the Seventh International Leprosy Congress, recommended the search for tissue cell systems that could substitute for natural hosts (Dhople, 1987). This recommendation stemmed from the belief that *M. leprae* is an intracellular obligate pathogen, with its main host cells having been identified as macrophages and Schwann cells; nevertheless, several authors, like Job et al., have described intracellular parasitism of the leprosy bacillus in macrophages, lymphocytes, hepatocytes, adrenal cortical and medullary cells, muscle cells, and endothelial cells of nine-banded armadillos (Job et al., 1989). As such, numerous cell lines have been investigated as potential tissue culture media for *M. leprae*.

#### 2.2.1 Macrophage cell cultures

Macrophages, as one of the primary host cells of *M. leprae*, have been the focus of efforts to develop macrophage-based culture systems for the growth of the leprosy bacillus.

##### 2.2.1.1 Mouse-derived macrophages

Mouse peritoneal macrophages were incubated in medium 4:5:1, previously reported to promote a faster growth rate of *M. lepraemurium*. Cultures of macrophages maintained in medium 4:5:1 supplemented with liver extract L fraction (1 mg/ml) and ferric nitrate (2 μg/ml), when incubated at 37 °C in an atmosphere of a 5% CO_2_–air mixture, showed an increase in macrophages numbers with the accumulation of inactive cells on top of the monolayer, while maintenance at 30 °C preserved the monolayer in good condition for weeks. Growth of AFB was observed in cultures inoculated with infectious material from lepromatous patients, with some cultures showing an increase in the average numbers of bacilli per macrophage from 12.0 to 28.2 at the end of 10 weeks. Afterwards, the organisms remained stable until the 22^nd^ week, when the cultures deteriorated. Identification of the cultured organisms was not reported in the manuscript (Chang and Neikirk, 1965).

Macrophages derived from MFP cells have also been studied as possible cell cultures for *M. leprae* growth. Using Eagle’s minimum essential medium (MEM) containing 10% FCS, an inoculated monolayer of MFP growth incubated at 25, 31, 33, and 37 °C for 72 and 96 h achieved an AFB recovery rate of 31.2% at 37 °C, 38.4% at 33 °C, 22.8% at 31 °C, and 15.4% at 25 °C. When MFP macrophages were incubated with L-15 (Leibovitz) medium supplemented with FCS (2%) at 30 °C, *M. leprae* survived for at least 54–70 days in some cultures, showing a certain increase in five out of fourteen experiments. However, all subcultures showed no evidence of growth, and AFB harvested did not multiply in mouse footpads. Thus, it was concluded that the increase in the number of bacilli was probably due to the breakdown of bacterial clumps (Matsuo and Utsunomiya, 1976).

Peritoneal and bone marrow macrophages from nude mice were incubated in complete National Cancer Institute Tissue Culture (NCTC) 109 medium containing 40% horse serum, 2% beef embryo extract, and ampicillin (100 μg/ml), with bone marrow cell culture medium supplemented with 10 U/ml of preservative-free heparin. Cultures were incubated in 1 ml of their corresponding medium at 35 °C and 5% CO_2_, with media replacement every 14 days. *M. leprae* was inoculated through incubation of cultures with a bacterial suspension overnight. The majority of cultures were maintained for 100 days or more. Of 44 experiments, only in 14 was there evidence of an increase in bacillary counts ranging from 1.1- to 4.5-fold (mean of 2.5-fold), but this was thought to be unlikely to represent growth or division of *M. leprae*; more likely due to the breaking up of globi or to fragmentation of acid-fast rods. In some experiments, horse serum was replaced with 40% foetal calf serum, and some cultures were incubated at 30 °C, although neither intervention proved effective. All subcultures attempted showed a decrease in the numbers of *M. leprae* (Sharp and Banerjee, 1984).

In 1997, Osawa reported the successful *in vitro* growth of *M. leprae* in mouse peritoneal macrophages. Cells were initially suspended in 20% heat-inactivated horse serum and 80% Waymouth solution and incubated at 37 °C in a closed atmosphere for 15–18 h. Afterwards, *M. leprae* was added with fresh medium, and 5% normal mouse serum was used to encourage phagocytosis, incubating the mix for 14 h. Once infection was achieved, the supernatant fluid was substituted with a culture medium including 20% heat-inactivated horse serum. Under these conditions, apparent multiplication of *M. leprae* was reported inside macrophages, and coccal-shaped cluster formation on the surface of the culture flask suggested to the author extracellular multiplication. The author ascribed his seeming success to the quality of the horse serum, but without being able to offer an explanation for its relevance (Osawa, 1997a).

##### 2.2.1.2 Human-derived macrophages

###### 2.2.1.2.1 Derived from mononuclear human peripheral blood cells (leprosy patients)

Human macrophages have also been used for *in vitro* culture of *M. leprae*. Cells derived from mononuclear peripheral blood cells of lepromatous patients, incubated in McCoy’s medium supplemented with 30% pooled AB normal human serum at 37 °C for 2 h to allow cell adherence to coverslips, were incubated with bacilli at 31, 34, or 37 °C. Under these conditions, Drutz and Cline studied the incorporation of 10 μCi of ^3^H-thymidine by phagocytosed *M. leprae*, which demonstrated that the isotope was mainly co-located with globi in cultures incubated at 31 °C. However, the authors reported that AFB were only present in 1% of culture cells and could not reach a conclusion regarding the temperature influence due to the limited number of experiments performed (Drutz and Cline, 1972).

###### 2.2.1.2.2 Monocytes derived from human peripheral blood

Further experiments used macrophages derived from monocytes isolated from the peripheral blood of lepromatous leprosy (LL), tuberculoid leprosy (TT), or healthy individuals. Cells were suspended in 5 ml of Eagle medium, gassed briefly with pure CO_2_, and initially incubated at 37 °C, then inoculated with *M. leprae* on the second day of cultivation. During the experiment, the medium was changed initially every 20 days, and later on, every week or 2 weeks, and incubation temperatures were either 37 °C or 33 °C. The majority of cell cultures in these conditions did not survive beyond 60 to 80 days. In 49% of experiments, there was an increase in the total number of bacilli of 2- to 9-fold (Samuel et al., 1973).

No significant differences were observed between macrophages from the different patient populations. Weekly changes of medium compared to every 20 days did not have a significant influence on the outcome of the experiment. Finally, increases in AFB were observed to a similar extent at 33 °C and 37 °C (Samuel et al., 1973).

##### 2.2.1.3 Macrophage–*M. leprae* interactions

It has been reported that unstimulated macrophages (from patients without a *M. leprae*-specific T-cell immune response) are unable to eliminate leprosy bacilli, allowing their survival and proliferation (Sibley and Krahenbuhl, 1988a). When macrophages are activated with interferon (IFN)-γ prior to contact with the bacteria, they can efficiently kill the bacilli; yet *M. leprae*-burdened macrophages are defective in responding to activating signals, including IFN-γ. This induced unresponsiveness has been extensively studied and has been proposed to be due to a reduction of macrophage protein metabolism (Salgame et al., 1980), to a reduced expression of surface receptors and sialic acid residues, or to the inhibitory effects of prostaglandin E_2_ (PGE_2_) secretion (Sibley and Krahenbuhl, 1988b). Understanding the causes of this effect could aid in reproducing it *in vitro* and allow *M. leprae* to develop unperturbed within macrophages.

###### 2.2.1.3.1 Suppression of macrophage activity by PGE_2_

Sibley and Krahenbuhl characterized this mechanism by incubating *M. leprae*-infected MFP granuloma macrophages in modified RPMI 1640 medium (Sibley and Krahenbuhl, 1988a). Under these conditions, granuloma macrophages produced 20-fold more PGE_2_ than normal peritoneal macrophages; after stimulation with IFN-γ and phorbol myristate acetate (PMA), increased O_2_^−^ production only occurred in peritoneal macrophages, maintaining granuloma macrophages’ baseline levels (Sibley and Krahenbuhl, 1988a). While co-culture of macrophages with 1.0 μg/ml of indomethacin preserved the response to IFN-γ (Sibley and Krahenbuhl, 1988b), once stablished, the lack of response could not be reversed by indomethacin despite reducing PGE_2_ production (Sibley and Krahenbuhl, 1988a). This suggests that the prolonged presence of PGE_2_ is not required or the non-prostaglandin mediated mechanisms may also be in effect.

The burst of PGE_2_ production took place on days 2–3 after infection with viable *M. leprae* of cultured peritoneal mouse macrophages incubated in the modified RPMI 1640 medium (Sibley and Krahenbuhl, 1988b). This timing is further corroborated by the fact that the response to IFY-γ stimulation is preserved in heavily burdened *M. leprae*-infected macrophages only when it is initiated 24 h following infection; if delayed until 5 days after infection, activation is inhibited (Sibley and Krahenbuhl, 1988b). The increase returned to baseline levels by day 7 of culture. Interestingly, Sibley reported that macrophages challenged with formalin-killed bacteria showed no change in PGE_2_ production, whereas pulsing with repeated doses of exogenous PGE_2_ for intervals of 3–4 days presented a similar effect to infection by *M. leprae* (Sibley and Krahenbuhl, 1988b).

###### 2.2.1.3.2 L-arginine metabolism

IFN-γ-activated macrophages act as the primary effector cells in the control of intracellular pathogens through their destruction by two products, the toxic radicals of oxygen and nitrogen; the latter of which appears to play a greater role in leprosy (Adams, 2021). The production of reactive nitrogen intermediates depends on L-arginine metabolism (the precursor molecule for a cytokine-inducible high output nitric oxide synthase), as several authors have reported that only in the presence of this amino acid can activated macrophages suppress the metabolic activity of *M. leprae* (measured by palmitic acid oxidation; Adams et al., 1991).

This has been demonstrated by incubating mouse peritoneal macrophages, some in supplemented phenol red-free Dulbecco’s modified Eagle medium (DMEM) containing 0.4 mM L-arginine and some in equally supplemented L-arginine-free RPMI 1640 medium (Adams et al., 1991).

When DMEM cultures were supplemented with 100 μM of N^G^-monomethyl-L-arginine (N^G^MMA), an L-arginine analogue, the inhibitory activity of activated macrophages was almost completely abrogated. If the L-arginine concentration was increased (up to 4.8 mM), the inhibition was reversed (Adams et al., 1991). Thus, there is a competitive inhibition of macrophage microbicidal activity dependent on L-arginine, through compounds such as N^G^-monomethyl-L-arginine (L-NMA), aminoguanidine (AG), and L-N^6^-(1-iminoethyl)-lysine (L-NIL; Adams, 2021). Furthermore, when 20 U/ml of arginase was added to the medium, macrophages were unable to inhibit *M. leprae* metabolism (Adams et al., 1991).

###### 2.2.1.3.3 Prior activation of macrophages

The relevance of prior activation of macrophages for *M. leprae* clearance was demonstrated by Hagge et al., who cultured bone marrow macrophages in Dulbecco’s modified Eagle medium (DMEM), supplemented with 5 μg/ml monocyte-colony-stimulating factor (M-CSF), and incubated at 37 °C until *M. leprae*-infected macrophages were added; when cultures were maintained at 33 °C (Hagge et al., 2004). Evaluating bacterial metabolic activity, a significant increase in activity when infected macrophages were co-cultured with non-activated macrophages was reported, whereas a decrease occurred when co-cultured with activated macrophages (Hagge et al., 2004). The authors suggested that a succession of challenges with unstimulated macrophages could potentially sustain *M. leprae* viability *in vitro* for prolonged periods of time (Hagge et al., 2004). This is further supported by the experiments of Adams et al., in which *M. leprae* showed high viability when recovered from infected granuloma macrophages cultured either alone or in the presence of non-stimulated macrophages, while bacteria were killed when cells were cultured with IFN-γ-activated macrophages (Adams, 2021). Thus, in agreement with Hagge et al. (2004), Adams et al. suggested that in the absence of cell-mediated immunity, the influx of fresh macrophages into a lepromatous leprosy lesion sustains the growth of bacilli; conversely, if effector macrophages are activated beforehand, *M. leprae* is killed when acquired from infected cells (Adams, 2021).

###### 2.2.1.3.4 *In vivo*-infected macrophages

Due to the long-term intracellular survival of *M. leprae* in macrophages *in vivo* during infection, compared to the short viability observed in macrophage cultures, some authors have suggested that during infection *in vivo* some factors must intervene that inhibit the macrophage bactericidal activity and which do not occur *in vitro*. Thus, attempts to culture *in vivo*-infected macrophages have been performed. Moura et al. cultured infected macrophages derived from multibacillary leprosy patients in 2 ml of modified RPMI 1640 medium, incubated at 37 °C in a humid atmosphere with 5% CO_2_ for up to 28 days, with media replacement every 2 days (Moura et al., 2007). The authors reported a progressive increase in the number of macrophages and nitrite levels in supernatant fluid, with a decline of infected macrophages from 35% on day 1 to 4% on day 24. These results suggest that multibacillary macrophages appeared able to eliminate *M. leprae in vitro* (Moura et al., 2007).

###### 2.2.1.3.5 Effects of IL-10 and/or TGF-β

To enhance the survival of *M. leprae* in cultured macrophages, culture media have been supplemented with different components, such as interleukin (IL)-10 or transforming growth factor β (TGF-β; Fukutomi et al., 2004), due to their immunomodulating effects.

When mouse peritoneal macrophages were maintained *in vitro* as described by Adams et al. (1991), cultures were infected with *M. leprae* and incubated in 1.0 ml media supplemented with the appropriate cytokine in 5% CO_2_ (Fukutomi et al., 2004). Bacterial metabolism declined rapidly in cultures incubated at 37 °C, while it increased for 15 days and maintained signs of viability after 25 days in cultures incubated at 31 °C. Supplementation with 2 U/ml of IL-10 prolonged *M. leprae* viability and allowed elongation of bacilli, while TGF-β had no effect (Fukutomi et al., 2004). The effects of IL-10 were ascribed to downregulation of innate production of baseline levels of reactive nitrogen intermediates (RNI), which possess strong anti-bactericidal properties, in non-activated macrophages. The authors considered that while baseline production was insufficient to kill bacilli, it could compromise their long-term viability (Fukutomi et al., 2004).

In a model of *M. leprae* granuloma, Wang et al. suggested that macrophages differentiated from peripheral blood monocytes using M-CSF, rather than granulocyte/monocyte (GM)-CSF, had a higher production of IL-10 in response to *M. leprae* infection (Wang et al., 2013), which could prove of interest bearing in mind the previous observations.

###### 2.2.1.3.6 Effects of IGF-I

Another molecule suggested to promote intracellular parasitic survival is insulin-like growth factor I (IGF-I), due to its ability to induce arginase activity, suppressing nitric oxide (NO) production as shown by Adams et al. (1991). Human monocytes cultured in modified RPMI 1640 medium, incubated at 37 °C and 5% CO_2,_ were able to produce IGF-I in response to *M. leprae* infection. Furthermore, supplementation of culture media with IGF-I reduced IFN-γ-induced NO production by 28% and induced IL-10 production in macrophages (Batista-Silva et al., 2016).

#### 2.2.2 Schwann cell cultures

The leprosy bacilli have the unique ability among mycobacteria to invade the peripheral nervous system. *M. leprae* has the capacity to cross the basal lamina surrounding the Schwann cell–axon unit and invade the cells. Binding of bacilli to the basal lamina is sufficient to engulf the bacterium, and it is mediated by the presence of the DNA-binding protein HU (or laminin-binding protein) and the cell wall antigen phenolic glycolipid 1 (PGL-1; Rambukkana, 2001). Once *M. leprae* adheres to the Schwann cell surface, they are internalized; however, the cells appear incapable of destroying intracellular bacilli (Hagge et al., 2002).

Schwann cells do not assemble a basal lamina when cultured in media lacking ascorbic acid or in the presence of *cis*-4-hydroxy-L-proline (a biosynthetic inhibitor of collagen formation; Einheber et al., 1993). Furthermore, the laminin α2 chain is thought to be secreted by Schwann cells only when they are grown in combination with neurons (Rambukkana, 2001). This could limit the use of Schwann cell monocultures. Nevertheless, other authors have reported that isolated Schwann cells constitutively synthesize laminin, and axonal contact only influences the distribution of laminin on the cell surface (a patchy expression in its absence, and a highly uniform distribution when axonal contact is present; Einheber et al., 1993).

Purified PGL-1 was found to bind specifically to the laminin α2 chain. This could explain the restricted tissue targeting of leprosy bacilli to Schwann cells. ML-LBP21 (a 21 kDa *M. leprae* laminin binding protein) also binds to peripheral nerve laminin-2, but it probably behaves as a common laminin-binding protein contributing to bacterial internalization without any tissue selectivity (Rambukkana, 2001).

*M. leprae* avidly binds to both myelinating and non-myelinating Schwann cell–axon units of Schwann cell–dorsal root ganglia (DRG) neuron co-cultures after 1 h of incubation (Rambukkana et al., 2002). It has been suggested that *M. leprae* is able to induce a non-immune-mediated demyelination of Schwann cells through PGL-1, facilitating cellular invasion, as myelinated Schwann cells appeared to better resist AFB invasion after 72 h (Rambukkana et al., 2002). Furthermore, *M. leprae* has been reported to induce nerve injury, causing Schwann cell proliferation, thus increasing the number of non-myelinating Schwann cells (Rambukkana et al., 2002).

An organized nerve culture of mouse sensory ganglia enriched for Schwann cells with cytosine arabinoside and maintained in 70% Dulbecco’s modified Eagle medium was developed by Mukherjee and Antia (Mukherjee and Antia, 1985). After inoculation with *M. leprae*, cultures were maintained at 37 °C with media changes twice a week. In this model, there was a gradual increase in AFB numbers per Schwann cell, peaking on day 28 post-inoculation (up to 10- to 12-fold) and declining afterwards (Mukherjee and Antia, 1985). This intracellular growth was transferable to subcultures, with a similar pattern. The authors concluded that *M. leprae* could be continuously subcultured without cross-contamination within Schwann cells (Mukherjee and Antia, 1985).

Schwann cells have also been maintained in modified minimal essential medium (MEM); in some cases, cultures were further supplemented with glial growth factor (GGF) and 2 μM forskolin (Einheber et al., 1993). To eliminate non-neuronal cells, alternate feedings of 5-fluorodeoxyuridine and uridine (10^−5^ M) were performed for 2 weeks (Einheber et al., 1993). However, the resultant cultures were not evaluated for their capacity to maintain *in vitro M. leprae* viability.

A further Schwann cell culture used for *M. leprae* growth was developed using glial cells isolated from the sciatic nerves of neonatal rats (Hagge et al., 2002). To enhance Schwann cell growth in these cultures, cells were mitotically expanded using β-heregulin (2.5 nM), forskolin (2 μM), and bovine pituitary extract (20 μg/ml). Heregulin was later removed from the medium 4 days prior to inoculation, while the latter were maintained throughout the experiment. After a confluent monolayer was achieved with this method, cultures were incubated for 24 h at either 33 or 37 °C in 5% CO_2_ prior to inoculation. No difference in cellular survival was observed at either temperature (Hagge et al., 2002).

When Schwann cells were infected, morphological alterations appeared within 4–12 days post-inoculation only after infection with viable *M. leprae* at 33 °C, with no change observed at 37 °C or when irradiated bacteria were used. Furthermore, in contrast to claims by Rambukkana et al. (2002) both myelinated and non-myelin-forming cells became infected. This discordance was attributed to technical differences, as multiplicity of infections (MOIs) < 100 and exposure times <48 h resulted in a lower percentage of infection in Schwann cells in the experiment by Hagge et al. (2002).

Hagge et al. reported that *M. leprae* retained 56% of the initial viability up to 21 days after infection in Schwann cell monolayers maintained at 33 °C, compared to only 3.6% at 37 °C. When cultures at 33 °C were maintained for 28 days, a 5% increase in metabolic activity assessed by radiorespirometry was observed between days 21 and 28. No data from long-term survival in cultures was reported as all experiments were terminated at 28 days post-inoculation (Hagge et al., 2002).

In parallel to data obtained from macrophage cultures, Schwann cells have also been reported to produce PGE_2_ and IL-10 48 h after *M. leprae* infection, with lower levels of IL-12 and NO compared with basal levels (Mattos et al., 2011).

#### 2.2.3 Minced chick embryo

McKinley and Verder reported successful cultivation of *M. leprae*, previously decontaminated with 3% NaOH, in minced chick embryos 7 to 11 days old, washed and suspended in Tyrode’s solution. Growth was obtained within 5 days under CO2 and O2 tension as well as under ordinary atmospheric conditions (McKinley and Verder, 1933). The authors also reported similar results with the use of human embryonic tissue and later successful culture of bacteria on solid media (such as hormone glycerol agar), appearing as discrete micro-colonies of acid-fast organisms (Mckinley and Vebder, 1933).

Hanks attempted a similar experiment inoculating chick embryos with leprosy nodule-derived material. Half the experiments were cultured at 34 °C, with the rest incubated at 37 °C. No growth was observed in any embryo after 16 days of incubation (Hanks, 1947a). This was in accordance with the findings of other researchers, who reported no bacterial growth after injection of AFB into the yolk sac of 5-day-old embryonated eggs (Embil et al., 1954).

#### 2.2.4 Breast muscle

Breast muscle from 10- to 15-day-old chick embryos, maintained in combinations of chicken serum with Simm’s serum ultrafiltrate, with fresh or pasteurized embryo juice, has been employed for *M. leprae* culture (Hanks, 1947a). Medium was renewed every 2–3 days, 7 days or 60–90 days, respectively. Cultures maintained at 37 °C with embryo juice induced such rapid growth of host cells that bacilli rapidly disappeared by dilution. Incubation at 34 °C slowed the growth rate significantly, and incubation at room temperature (29–31 °C) allowed cultures to be maintained without the need for medium renewal for 90 days. Nevertheless, leprosy bacilli showed no signs of proliferative activity (Hanks, 1947a).

#### 2.2.5 Other cellular cultures

Other tissues have been explored for the *in vitro* growth of *M. leprae*.

A review by Pattyn (1973) included references to several works, such as Shepard (Shepard, 1958), who reported no multiplication of mycobacteria in human and monkey tissue cells; Lagoa (original manuscript unavailable), who observed intracellular growth of fuchsinophilic granules inside monkey kidney tissue cells; Delville (original manuscript unavailable), who described limited multiplication in cultures of human amniotic cells and promising results in cell cultures derived from Kaposi’s sarcoma; and Ranadive et al. (1958), who had no success with cultures of human foetal spinal ganglion cells.

Fieldsteel and McIntosh reported on an experiment evaluating various tissue culture systems for *M. leprae* (Fieldsteel and McIntosh, 1972). Tissues investigated included mouse testis and eye, rat testis, and human foreskin, embryonic skin-muscle, embryonic fingertip, amnion, leukocytes, sarcoma, and leproma. Cultures were incubated at both 31 and 34 °C. Initially, Eagle’s minimum essential medium supplemented with 5% inactivated FBS and 100 U/ml penicillin was used under conditions of 100% humidity and 5% CO_2_. In subsequent experiments, L-15 medium containing 10% inactivated FBS was employed under free gas exchange conditions and 70% humidity, while leukocytes were maintained in medium 199 supplemented with 20% FBS. Nutrient media were replaced when excessive acidification occurred, when signs of cytotoxicity became apparent, or when cell sheets began to detach from the glass (for leukocyte cultures, medium changes were performed every 55 to 93 days).

In these experiments, the tissues with the highest phagocytic activity were human leukocytes (100%), human leproma (84%), and mouse testis (70%). At 34 °C, maximum phagocytosis was reached after 2 weeks, whereas at 31 °C, phagocytosis was slower (2 weeks) and reached slightly lower levels. After incubation, no instance of *M. leprae* multiplication had occurred. Viability of leprosy bacilli was maintained for 118 days in mouse testis and 64 days in human leukocytes. The rest of the tissues examined eliminated the AFB at earlier points during incubation (Fieldsteel and McIntosh, 1972).

Hanks also evaluated bacillary growth in fibroblast tissue cultures from lepromatous lesions in serum media, failing to observe any increase in the total mass of bacilli (Hanks, 1947b). The author commented that although the number of bacilli or the proportion of bacillated cells may rise for a time, this was mainly caused by technical factors or because the microorganisms were in reality derived from the explants. After intervals of 60 to 90 days, the proportion of infected cells declined steadily. Hanks added that supplementation of culture medium with embryo juice did not have significant effects on tissue cultures. Incubating temperatures of 34 °C, slow culture growth, low cell metabolism, and a slightly alkaline medium were the combination that maintained bacilli in the cells for the longest interval (Hanks, 1947b).

#### 2.2.6 Complex tissue cultures

Complex tissue cultures have been used in an effort to simulate *in vivo M. leprae* environment. Human organotypic skin explant culture (hOSEC) has been studied by de Paula N A *et al.* for its similarities with *in vivo* human skin (De Paula et al., 2024). Skin tissue samples are manipulated inside a laminar flow hood and placed in phosphate-buffered saline (PBS, pH 7.2) plus 1.5% antibiotic solution (100 U/ml penicillin and 100 mg/ml streptomycin) overnight at 4 °C for decontamination. Then, subcutaneous tissue is removed with scissors and full-thickness skin explants are placed with the dermal side facing down on pieces of filter paper (80 g/m^2^, 26 L/s m^2^ air permeability, 25 μm porosity) supported by metal grids. Explants are incubated in 5 ml of supplemented DMEM, with 2 ml of exhausted medium replaced every third day.

Under these conditions, Paula inoculated each explant with a suspension of *M. leprae* of 86% viability and incubated cultures at 37 °C in 5% CO_2_ for 4, 7, 14, 28, and 60 days (De Paula et al., 2024). Viability results were reported as maintenance of cycle thresholds of 16S rRNA by RT-PCR throughout cultivation. After *ex vivo* cultivation, bacilli were extracted and inoculated in nude mice. Sixty percent of animals inoculated with 28-day-old (D28) bacilli and 42.9% inoculated with 60-day-old (D60) bacilli showed amplification of 16S rRNA after 5 months; mice receiving suspension from D28 showed 27% positive microscopy for AFB by Ziehl–Neelsen (ZN) compared to 17.9% that received from D60.

##### 2.2.6.1 Free-living amoeba

It has been reported that parasites such as *Acanthamoeba castellanii* and *A. polyphaga* are capable of taking up *M. leprae* bacilli (Lahiri and Adams, 2016). Intracellular bacilli are subsequently enclosed within a single large vacuole, creating an acidic environment similar to that found in macrophages. In this way, bacilli are able to survive in dormant encysted amoebae. To date, no amoeba cell cultures of *M. leprae* have been published.

#### 2.2.7 Tick cell lines

After the identification of several animals, including the armadillo *Dasypus novemcinctus* (Truman et al., 2011), non-human primates (Gormus et al., 1988; Honap et al., 2018), and red squirrels (Avanzi et al., 2016) as potential reservoirs of *M. leprae*, the implication of hematophagous arthropods in leprosy transmission has been investigated (Ferreira et al., 2018). Ticks, particularly those from the genus *Amblyomma* have emerged as the most promising candidates (de Souza-Araujo, 1941).

To evaluate the potential of *Amblyomma sculptum* ticks as leprosy vectors, Ferreira et al. (2018) studied the growth of *M. leprae* in embryonic or larval cells from different tick species (*Amblyomma* var*iegatum, Hyalomma anatolicum*, and *Ixodes scapularis*) *in vitro*. Multiplication of *M. leprae* was demonstrated through the 16S rRNA qPCR technique in *I. scapularis* embryonic (IDE8) cells incubated in L-15B medium (Munderloh and Kurtti, 1989) supplemented with 10% FBS, 10% tryptose phosphate broth (TPB), 0.1% bovine lipoprotein–cholesterol concentrate and 2 mM L-glutamine at 30 °C. Cells were subjected to infection with *M. leprae* Thai-53 strain at a MOI of 50 bacteria per cell (Ferreira et al., 2018).

The authors reported a detectable increase in the number of bacilli over a 20-day period, an *in vitro* doubling time of approximately 12 days, and maintenance of virulence demonstrated by the MFP technique. Bacterial viability after 59 days *in vitro* was estimated to range between 70 and 90% using the LIVE/DEAD staining method. Lastly, the authors report that a protocol for continuous *in vitro* cultivation of *M. leprae* is under development (Ferreira et al., 2018).

## 3 Discussion

*In vitro* cultivation of *M. leprae* has remained an elusive achievement in the field of microbiology since its discovery. Early reports of successful cultivation of the leprosy bacilli (Delville and Pichel, 1975; Biswas, 1997) have later been attributed to environmental mycobacteria or species such as *M. scrofulaceum* (Bapat, 1989; Skinsnes et al., 1978). The characteristic growth pattern of *M. scrofulaceum* (slow, fastidious growth in primary cultures followed by rapid growth in subcultures) parallels the findings reported from these supposedly successful *M. leprae* cultivation experiments (Delville and Pichel, 1975; Biswas, 1997). Therefore, conclusions from the previously mentioned authors are expected not to apply to true *M. leprae in vitro* growth. On the other hand, recent reports of *M. leprae* growth in cell media (De Paula et al., 2024; Ferreira et al., 2018) need to be confirmed in further experiments, and long-term and sustainable growth remains to be evaluated.

One major challenge in cell culture studies is that many cell lines proliferate faster than the slow-growing leprosy bacillus, overgrowing the culture and displacing infected cells. It is generally accepted that no useful result is attainable from work with rapidly dividing cells that have a generation time shorter than that expected for *M. leprae*. Another problem is that some reported findings of replicating AFB may, in fact, be due to cell lysis and the release of bacilli taken up by other cells. Consequently, no successful tissue culture has yet been established that allows continuous multiplication of *M. leprae* (Lahiri and Adams, 2016).

Early experimental efforts focused on identifying metabolic limitations of the bacterium and supplementing axenic media accordingly. Nakamura concluded that metabolic supplementation was insufficient, positing that structural frailty of the bacterium, rather than metabolic deficiencies, is the key barrier to *in vitro* growth (Nakamura, 2001). This perspective aligns with genomic analyses showing that *M. leprae* retains most core biosynthetic pathways, being capable of synthesizing most of its own components (Wheeler, 2003).

The *M. leprae* genome is composed of 3.27 Mb (Silva et al., 2022), which makes it much smaller than the *M. tuberculosis* genome (4.4 Mb; Silva et al., 2022; Han et al., 2009); moreover, it contains approximately 1,600 pseudogenes (Singh and Cole, 2011) and has lost 50% of the genes of the last common ancestor with *M. tuberculosis*. This genome downsizing has been assumed to be the result of reductive evolution, probably due to adaptation to an intracellular parasitic lifestyle (Han et al., 2009). The recently described *M. lepromatosis* (Sharma et al., 2020) shares 90.9% homology, with similar metabolic functions but with a higher tendency to invade endothelial cells. *Mycobacterium haemophilum* is the closest mycobacterium phylogenetically related to the leprosy bacilli, but it has a genome consisting of 4.24 Mb, almost all functional coding genes, and retains the ability to grow *in vitro* (Silva et al., 2022; Han et al., 2009).

Genomic analyses have reported that around half of the genes related to energy metabolism turned out to be pseudogenes (Cole et al., 2001). For instance, the pyruvate carboxylase gene is lacking in *M. leprae*, and this is predicted to incapacitate the bacteria from using pyruvate for glycan biosynthesis while being able to utilize it for energy production and fatty acid biosynthesis. Furthermore, suspensions of *M. leprae* did not use acetate, nor were the enzymes for its use detected, and there were no active genes for the utilization of galactose (Wheeler, 2003). Nevertheless, an *in vivo* utilization of carbohydrates is thought to exist due to the identification of two integral membrane proteins involved in the translocation of sugars across the membrane, uspD and ML1425 (Marques et al., 2008). This could mean that *M. leprae* may require a mix of carbon sources to attain balanced growth (Wheeler, 2003).

Although the enzymes involved in glycolysis, the pentose cycle, and gluconeogenesis are all complete in the leprosy bacilli (Wheeler, 2003), the concentration of intermediate metabolites related to central carbon metabolism (such as glucose-6-phosphate, fructose-6-phosphate, sedoheptulose-7-phosphate, 6-phosphogluconic acid, and ribose-5-phosphate) were 0.1-fold found in *M. leprae* compared to *M. bovis* (Miyamoto et al., 2016). These suggested a declined or repressed state of central carbon metabolism in leprosy bacilli.

In an analysis of bacterial metabolites, it was found that 69% of the total intracellular metabolites of *M. leprae* were from amino acid metabolism, with a lesser proportion of metabolites associated with central carbon and nucleic acid metabolism. This was ascribed by Miyamoto *et al.* to either a high bacterial production capacity of amino acids or a high uptake of host-derived amino acids (Miyamoto et al., 2016).

Lipid metabolism plays a key role in intracellular survival. Marques *et al.* reported that the enzymes for fatty acid *β*-oxidation were located in association with the membrane compartment, reinforcing the authors’ theory that fatty acids, rather than carbohydrates, are the dominant carbon substrate utilized by mycobacteria during infection. This would be further supported by the utilization of an active glyoxylate cycle, the dominant anaplerotic pathway during growth on fatty acids. Two enzymes involved in it, isocitrate lyase and malate synthase, were present in the proteomic profiles of the cell wall and membrane (Marques et al., 2008). Furthermore, deletions of genes encoding proteins utilizing lipids as a sole carbon source rendered *M. leprae* incapable of growth *in vivo* (Mattos et al., 2012).

*M. leprae* has 11 active *fadD* genes, responsible for fatty acid scavenging (mechanisms used by pathogenic mycobacteria to convert free fatty acids into fatty acyl-CoAs, bypassing the limitations of acetyl-CoA production via glycolysis; these fatty acyl-CoAs can then be metabolized and used for the synthesis of endogenous fatty acids and mycolic acids, crucial to the integrity of all mycobacterial cells), while only sporting 2 *lip* genes (encoding lipase enzymes; Wheeler, 2003); this lipase deficiency has been theorized to be compensated by the increased expression of lipase genes in infected host cells (Mattos et al., 2011). *M. leprae* has also lost most of the genes associated with cholesterol catabolism, retaining only the ability to oxidize cholesterol to cholestenone (Marques et al., 2008). Thus, although cholesterol appears to play an important role in intracellular survival (Lobato et al., 2014), the lipids found in infected host cells are derived from the host lipids.

Identification of the enzymes alcohol dehydrogenase and lactate dehydrogenase confirms that *M. leprae* relies on alcohol and lactate fermentation as an alternative pathway to regenerate NAD^+^, due to the loss of codifying genes of the NADH dehydrogenase. Respiration is active, as all the subunits of ATP synthase are detected, and the pentose phosphate and citric acid cycle pathways are also functional (Marques et al., 2008). However, the leprosy bacilli respiratory pathway has FADH as the single electron donor, and pyruvate ingress to the Krebs cycle is limited by the presence of a single pyruvate dehydrogenase complex (Wheeler, 2003).

In *M. leprae* there is a single copy of the diguanylate cyclase/phosphodiesterase gene compared to the three copies present in *M. lepromatosis*. This enzyme has two antagonistic domains, involved in the synthesis and hydrolysis of the second messenger cyclic di-GMP. This cyclase has a potential role in the signalling response to intracellular survival, as its deletion in *M. tuberculosis* affected its dormancy and pathogenicity (Silva et al., 2022).

*M. leprae* also expresses enzymes involved in the synthesis of amino acids (such as threonine, cysteine, lysine—although some authors have reported *M. leprae* to be a lysine auxotroph (Ojo et al., 2022)—aspartate, proline, histidine, valine, isoleucine, leucine), folic acid, riboflavin, thiamine, heme, and purine and pyrimidine nucleotides (Marques et al., 2008). The genome lacks most of the genes for cobalamin (vitamin B12) biosynthesis, with only the genes that encode enzymes that have adenosine or its nucleotides as a substrate persisting; thus, the required vitamins would have to be included in any culture medium (Wheeler, 2003).

Although *M. leprae* has been reported to lack *metC* and other genes involved in sulphate transport, suggesting a lesion in methionine biosynthesis (Wheeler, 2003), later studies (Ojo et al., 2022) have discovered an alternative pathway for methionine biosynthesis. Moreover, neither the lesion in cobalamin nor methionine biosynthesis accounts for the failure to grow *M. leprae* in culture media, as both compounds are frequently included in common mycobacterial culture media.

Some authors have reported a short-lived *M. leprae* growth *in vitro*, followed by rapid degeneration of bacilli or by the inability of cultured bacteria to continue growing on subcultures. This has been attributed to a supposed host-dependent factor (Baker, 1983; Nakamura, 1995b). Host-derived (lysosomal) hydrolases have been reported to be tightly bound to the bacterial surface, presumably participating in the hydrolysis of environmental substrates, complementing mycobacterial metabolism (Delville and Pichel, 1975). Could these enzymes be the famous host-derived factor involved in early *in vitro* growth?

As *M. leprae* has long been regarded as an obligate intracellular pathogen, great efforts have been employed to thoroughly characterize its intracellular niche. Infected macrophages and Schwann cells from lepromatous leprosy patients have been described to accumulate large amounts of lipids. Although initially thought to be derived from *M. leprae* metabolism, further analysis demonstrated that the foamy aspect is due to the accumulation of host-derived lipids, such as triacylglycerol, oxidized phospholipids, free cholesterol, and cholesterol ester (Mattos et al., 2011; Cruz et al., 2008; Mattos et al., 2010). These lipids are stored in non-membrane-bound cytoplasmic organelles known as lipid bodies or lipid droplets (LDs; Mattos et al., 2010; Elamin et al., 2012).

LDs formed in response to *M. leprae* constitute sites for eicosanoid synthesis, being responsible for the increased production of PGE_2_ previously described (Mattos et al., 2010). Toll-like receptor (TLR)2 and TLR6 pathways are preferentially activated during LD biogenesis triggered by *M. leprae* infection in macrophages (Mattos et al., 2010), while TLR6 is the critical pathway in Schwann cells (Mattos et al., 2011). Moreover, in glial cells, LD formation is only induced by live bacteria, unlike in macrophages, in whom non-viable bacilli or mycobacterial glycolipids are sufficient (Mattos et al., 2011).

LDs are relevant, as they have been described to play a role in *M. leprae* intracellular survival, as a decrease in bacterial viability was observed upon inhibition of LD biogenesis (Mattos et al., 2011). LDs are promptly recruited to and accumulate in bacteria-containing phagosomes (Mattos et al., 2012; Mattos et al., 2011). They have been suggested to act as a rich source of nutrients for mycobacterial intracellular growth (Mattos et al., 2011). In addition, macrophage LDs have been shown to accumulate mycobactin (the lipophilic siderophore of mycobacteria)–iron complexes, and ferric mycobactin-enriched LDs are found in close contact with phagosomes (Mattos et al., 2012). This suggests that foamy bodies enable the delivery of iron to the mycobacteria.

**Figure 1:**
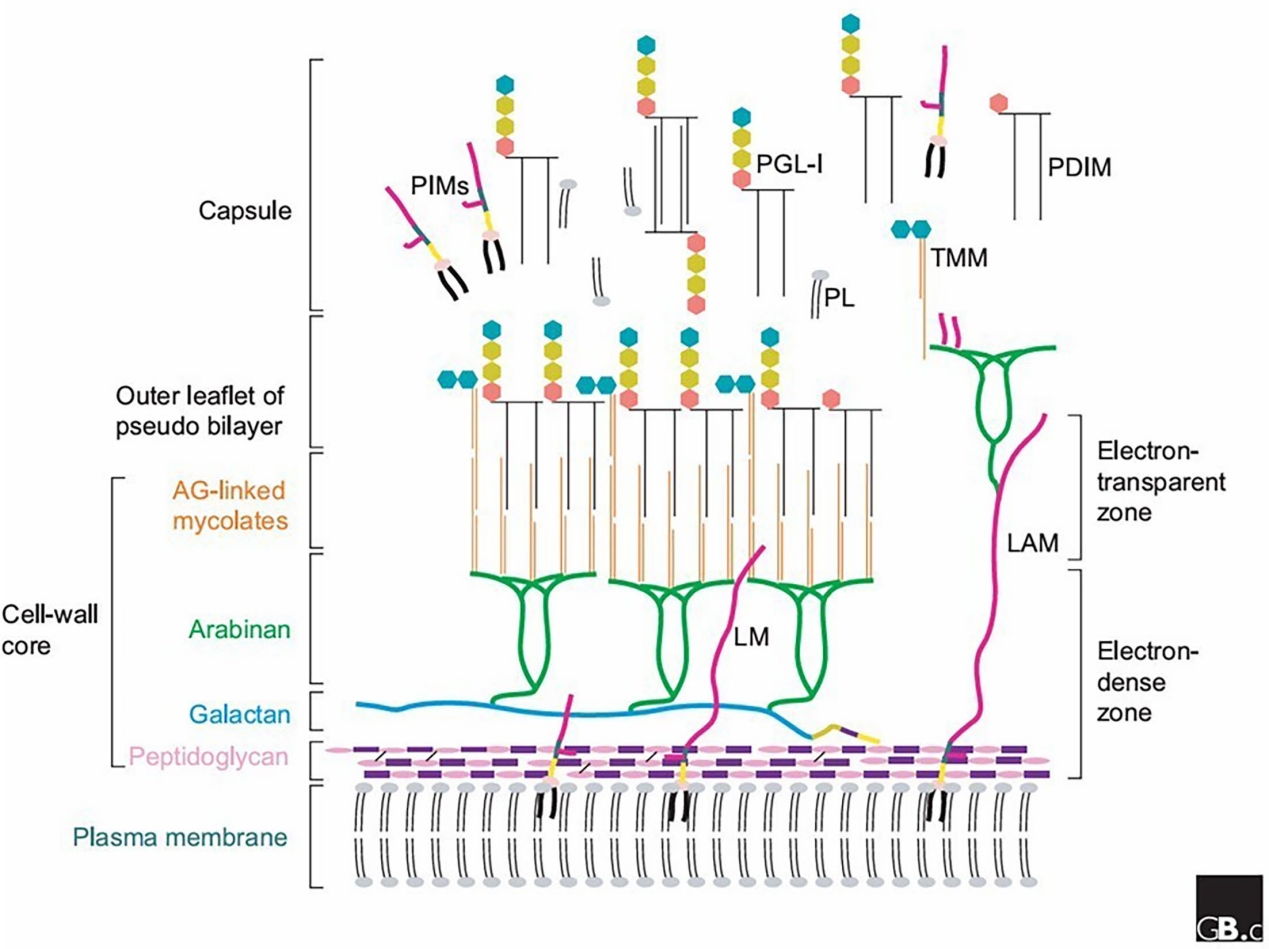
The plasma membrane is covered by a cell-wall core made of peptidoglycan (chains of alternating GlcNAc and MurNGly, linked by peptide crossbridges) covalently linked to the galactan by a linker unit (-P-GlcNAc-Rha-) of arabinogalactan. Three banched chains of arabin are in turn linked to the galactan. The peptidoglycan-arabinogalactan layer forms the electron-dense zone. Mycolic acids are linked to the termini of the arabinan chains to form the inner leaflet of a pseudo lipid bilayer. An outer leaflet is formed by the mycolic acids of TMM and mycocerosoic acids of PDIMs and PGLs as indicated. The pseudo-bilayer forms the electron-transparent zone. A capsule presumably composed largely of PGLs and other molecules such as PDIMs, PIMs and phospholipids surround the bacterium. Lipoglycans such as PIMs, LM and LAM, known to be anchored in the plasma membrane are also found in the capsular layer as shown. Abbreviations. GlcNAc: N-actulglucosamine; MurNGly: N-glycolylmuramic acid; P: Phosphate; Rha: Rhamnose; TMM: Trehalose monomycolate; PDIM: Phthiocerol dimycocerosate; PIM: Phosphatidylinositol mannoside; LM: Lipomannan; LAM: Lipoarabinomannan. Original figure from Vissa VD, Brennan PJ. The genome of Mycobacterium leprae: a minimal mycobacterial gene set. Genome Biol. 2001;2(8):1023.1-1023.8 (91).

Furthermore, Nakamura concluded his extensive experiments on *in vitro* growth of *M. leprae* with the claim that the inability of the leprosy bacillus to grow was not due to metabolic deficiencies but due to the frailty of its cell wall (Nakamura, 2001). The cell wall is mainly composed of mycolic acids and peptidoglycan (Figure 1; Vissa and Brennan, 2001); in the latter, the L-alanine of the common sequence alanyl-D-isoglutaminyl-meso-diaminopimelyl-D-alanine is replaced in *M. leprae* by glycine, which could explain its fragility (Draper, 1983). Thus, the author maintained that the strengthening of the bacterial cell wall and membrane was the prerequisite for a successful cultivation of the bacilli in axenic media (Nakamura, 2001). LDs may be responsible for this strengthening effect *in vivo*; therefore, the creation of a culture medium capable of packaging *M. leprae* in LD-like structures may enable bacilli *in vitro* growth.

In conclusion, we present the main axenic and tissue culture media developed to promote *M. leprae* survival and multiplication *in vitro*. Despite numerous attempts at axenic and tissue culture media, continuous *M. leprae* growth *in vitro* remains elusive. Genomic analysis suggests that metabolic insufficiency is unlikely to be the primary barrier; rather, structural fragility and the need for host-like intracellular environments may be the key. Moreover, microphysiological systems and organoleptic models open the opportunity for better exploration of *in vitro* growth of *M. leprae* as they may better simulate *in vivo* conditions. Further studies are needed to characterize the structural differences of the *M. leprae* cell wall compared to other mycobacteria and how these differences can impact its environmental resistance.

## 4 Materials and methods

We performed a descriptive bibliographic review of the literature following a strict methodology to guarantee transparency and rigor in the selection, evaluation and synthesis of included studies.

### 4.1 Search criteria

A literature search was conducted in May 2025 using the following electronic databases:

- PubMed: using MeSH terms and key words related to *M. leprae* cultivation, leprosy, and microbiology.
- Embase: search terms employed were similar to those used in PubMed, with an additional focus on preclinical studies.
- Cochrane Library: systematic reviews and randomized controlled trials related to leprosy and the cultivation of *M. leprae* were searched.

Within each database, our search strategy involved the use of free terms (leprosy, cultivation, *in vitro*), key words, and controlled terms such as Desc, MeSH, and the thesaurus Emtree, including terms describing the microorganism (*Mycobacterium leprae*), the clinical disease (leprosy), the techniques researched (*in vitro* techniques), as well as free terms (leprosy cultivation) and combining them using Boolean operators. The detailed search strategy is specified in Annex 2.

### 4.2 Inclusion and exclusion criteria

All articles reporting *M. leprae* cultivation were included, regardless of their design; articles not dedicated to *M. leprae* cultivation or which did not include a methodology related to *in vitro* culture, such as *in vivo* models, were excluded.

The search strategy included all articles without restriction of time period but was limited to Spanish, French, or English language, as these are the most common languages used in leprosy research. When articles were recovered, priority was given to original articles, reviews, systematic reviews, and meta-analyses, due to the more detailed and structured approach to the subject under revision.

### 4.3 Screening

References of recovered studies fulfilling inclusion criteria were reviewed to identify further articles with possible relevance to the present review.

After applying the prior filters, the studies were evaluated according to specific inclusion criteria, such as relevance to the research question, duplicates, or inaccessibility to the full text. Article titles and abstracts of the initial search results were screened to identify those appropriate for full-text review. In this first screen, those articles that did not meet inclusion criteria were eliminated.

Results of the initial screen were imported to a reference manager (Zotero 7) for duplicate elimination. The full text of identified articles after screening was recovered. In several studies, full text was not available after extensive research aided by the institutional library; those articles were excluded from further review, yet references to their authors are provided when historically relevant or when necessary for discussion of the results of available studies. Those studies for which a complete text was available were evaluated to ensure their relevance, methodological quality, and pertinence to the present theme.

**Figure 2:**
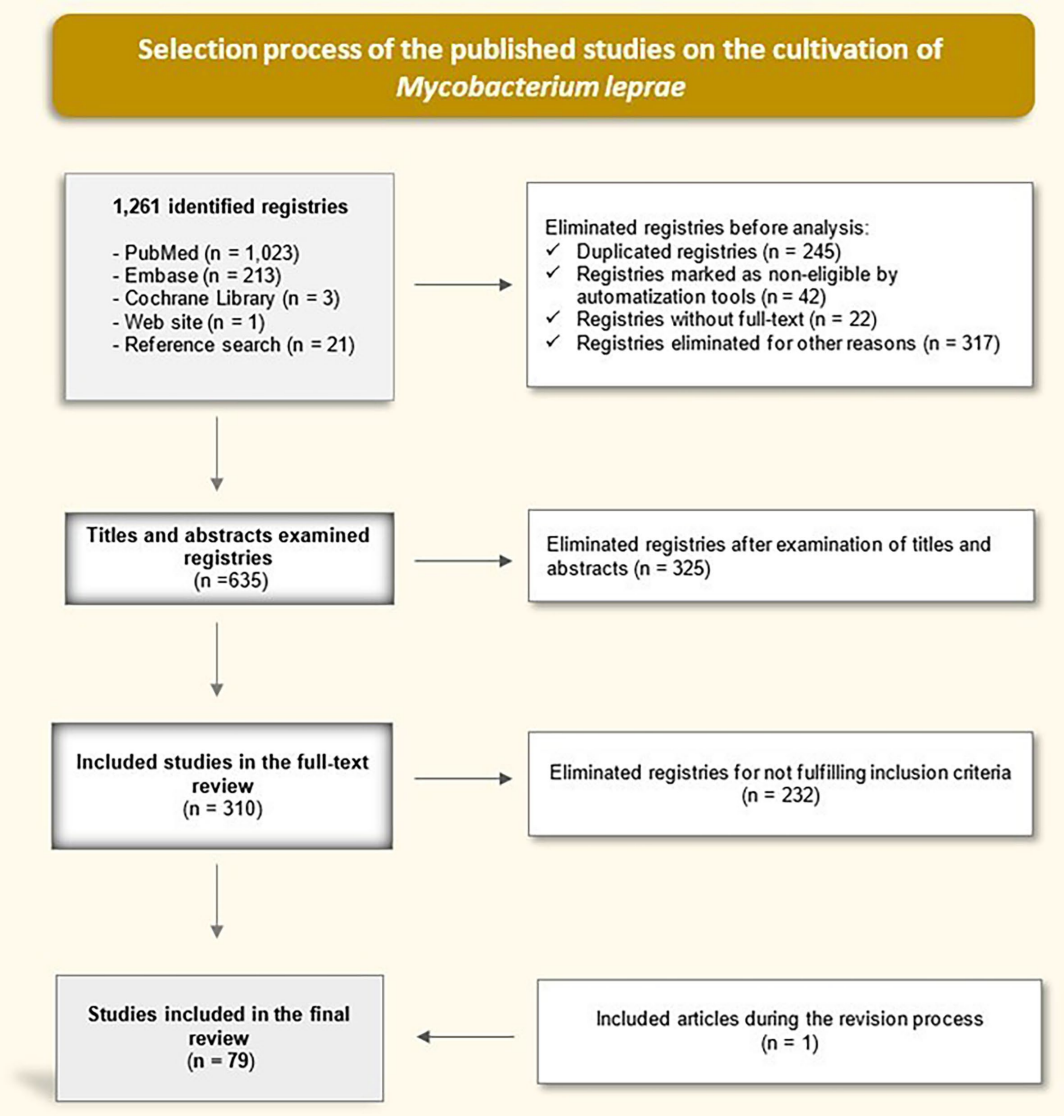
Flow chart of studies screened and included in this study.

The search and screening process was documented through the following diagram (Figure 2) for a visual description of each step of the process, including the number of articles initially identified, the number of articles eliminated, and the number of articles included in the analysis.

A total of 1,261 articles were recovered from the databases during the initial research. After the removal of duplicates (245) and records removed for other reasons (317; mainly conference reports without original text available), 635 unique records remained. During the screening phase, 325 articles were excluded after reviewing titles and abstracts, leaving 310 articles for full-text evaluation. In the last step, 232 articles were excluded, leaving 57 reports that fulfilled inclusion criteria. These articles were supplemented by 21 studies recovered from the review of the references of the previous reports and 1 article included during the review process. In total, 79 studies were included in the final analysis, covering works from 1933 to 2024.

## Data Availability

The original contributions presented in the study are included in the article/Supplementary material, further inquiries can be directed to the corresponding author.
