# Supplementary material for: Silence on the plate: revisiting the enigma of Mycobacterium leprae cultivation
Source: Front Microbiol. 2025 Dec 3;16:1708557. doi: 10.3389/fmicb.2025.1708557 (PMC12708612; doi:10.3389/fmicb.2025.1708557)
Supplement: Supplementary file 1 [file Data_Sheet_1.docx]

**ANNEX 1**

In the following list we present the culture media discussed in the review and indicate the number of times each culture medium has been evaluated. Furthermore, we indicate the main authors of the manuscripts in which the culture media were discussed.

**Axenic culture media**

- BME medium 🡪 1 (Delville and Pichel, 1975)
- RPMI-1640 medium 🡪 1 (Delville and Pichel, 1975)
- Dubos medium 🡪 3 (Delville and Pichel, 1975; Dhople *et al.*, 1988; Biwas, 1997)
- KI-1 medium 🡪 1 (Kato and Ishaque, 1977)
- LA-3 medium 🡪 2 (Skinsnes *et al.*, 1975; Dhople *et al.*, 1988)
- Modified Eagle’s medium 🡪 1 (Oltizki, 1977)
- NM3 medium 🡪 1 (Oltizki, 1977)
- MY 14b agar medium 🡪 2 (Nakamura *et al.*, 1982; Dhople *et al.*, 1988)
- Nutrient-Tween medium 🡪 1 (Lee and Colston, 1985)
- Thioglycollate medium 🡪 1 (Lee and Colston, 1985)
- DH medium 🡪 1 (Dhople *et al.*, 1988)
- Mahadeevan’s medium 🡪 1 (Dhople *et al.*, 1988)
- Middlebrook 7H9 🡪 1 (Dhople *et al.*, 1988)
- Wheeler’s medium 🡪 1 (Wheeler, 1988)
- Ishaque’s liquid medium 🡪 1 (Ishaque, 1990)
- Ishaque’s solid medium 🡪 1 (Ishaque, 1990)
- ML medium 🡪 1 (Osawa, 1997)
- NHDP medium 🡪 1 (Ojo *et al.*¸ 2022)
- NK-180 medium 🡪 1 (Nakamura, 1998)
- Kirchner medium 🡪 1 (Nakamura and Matsuoka, 2001)
- NK-260 medium 🡪 2 (Nakamura, 2001; Amako *et al.*, 2016)

**Cell culture media**

- Mouse-derived macrophages 🡪 4 (Chang and Neikirk, 1965; Matsuo *et al.*, 1976; Sharp and Banerjee, 1984; Osawa, 1997)
- Human-derived macrophages 🡪 8 (Drutz and Cline, 1972; Samuel *et al.*, 1973; Sibley and Krahenbuhl, 1988; Hagge *et al.*, 2004; Adams *et al*., 1991; Moura *et al.*, 2007; Fukutomi *et al.*, 2004; Batista-Silva *et al.*, 2016)
- Schwann cells 🡪 2 (Einheber *et al.*, 1993; Mukherjee and Antia, 1985)
- Complex tissue 🡪 1 (De Paula *et al.*, 2024)
- Tick cell lines 🡪 1 (Ferreira *et al.*, 2018)
