# Supplementary material for: Silence on the plate: revisiting the enigma of Mycobacterium leprae cultivation
Source: Front Microbiol. 2025 Dec 3;16:1708557. doi: 10.3389/fmicb.2025.1708557 (PMC12708612; doi:10.3389/fmicb.2025.1708557)
Supplement: Supplementary file 2 [file Data_Sheet_2.docx]

**ANNEX 2**

**PUBMED SEARCH STRATEGY:**

(((("Mycobacterium leprae"[MeSH Terms] OR "Leprosy"[MeSH Terms]) AND ("cultivability"[All Fields] OR "cultivable"[All Fields] OR "cultivate"[All Fields] OR "cultivated"[All Fields] OR "cultivates"[All Fields] OR "cultivating"[All Fields] OR "cultivation"[All Fields] OR "cultivations"[All Fields] OR "cultivator"[All Fields] OR "cultivators"[All Fields] OR ("in vitro techniques"[MeSH Terms] OR ("vitro"[All Fields] AND "techniques"[All Fields]) OR "in vitro techniques"[All Fields] OR "vitro"[All Fields] OR "in vitro"[All Fields]))) OR (("Mycobacterium leprae"[MeSH Terms] OR "Leprosy"[MeSH Terms]) AND "cultiv*"[All Fields]) OR (("Mycobacterium leprae"[MeSH Terms] OR "Leprosy"[MeSH Terms]) AND ("cultivability"[All Fields] OR "cultivable"[All Fields] OR "cultivate"[All Fields] OR "cultivated"[All Fields] OR "cultivates"[All Fields] OR "cultivating"[All Fields] OR "cultivation"[All Fields] OR "cultivations"[All Fields] OR "cultivator"[All Fields] OR "cultivators"[All Fields] OR ("in vitro techniques"[MeSH Terms] OR ("vitro"[All Fields] AND "techniques"[All Fields]) OR "in vitro techniques"[All Fields] OR "vitro"[All Fields] OR "in vitro"[All Fields])))) AND ("english"[Language] OR "french"[Language] OR "spanish"[Language]))

RESULTS: 1023

*MeSH Terms are listed prior to the reference “[MeSH Terms]”. Keywords and free terms are used indistinctly and identified by the reference “[All Fields]”.*

**COCHRANE LIBRARY SEARCH STRATEGY:**

Mycobacterium leprae OR leprosy

RESULTS: 3

**EMBASE SEARCH STRATEGY:**

('leprosy' OR 'mycobacterium leprae') AND ('cultivation' OR 'in vitro') AND ('article'/it OR 'review'/it) AND [embase]/lim NOT ([embase]/lim AND [medline]/lim)

RESULTS: 213
